# Kidney microbiota dysbiosis contributes to the development of hypertension

**DOI:** 10.1080/19490976.2022.2143220

**Published:** 2022-11-12

**Authors:** Xin-Yu Liu, Jing Li, Yamei Zhang, Luyun Fan, Yanli Xia, Yongyang Wu, Junru Chen, Xinyu Zhao, Qiannan Gao, Bing Xu, Chunlai Nie, Zhengyu Li, Aiping Tong, Wenjie Wang, Jun Cai

**Affiliations:** a State Key Laboratory of Biotherapy and Cancer Center, West China Hospital, Sichuan University, Chengdu, China; b Hypertension Center, State Key Laboratory of Cardiovascular Disease, National Center for Cardiovascular Diseases, Fuwai Hospital, Chinese Academy of Medical Sciences and Peking Union Medical College, Beijing, China; c Heart Center & Beijing Key Laboratory of Hypertension, Beijing Chaoyang Hospital, Capital Medical University, Beijing, China; d Clinical Genetics Laboratory, Affiliated Hospital &Clinical Medical College of Chengdu University, Chengdu, P.R. China; e School of Food and Biological Engineering, Chengdu University, Chengdu, China; f Department of Urology, Affiliated Sanming First Hospital, Fujian Medical University, Sanming, China; g Reproductive and Genetic Hospital of CITIC‐Xiangya, Changsha, China; h Department of Proctology, University of Chinese Academy of Sciences-Shenzhen Hospital (Guang Ming), Shenzhen, China; i Department of Obstetrics and Gynecology, Key Laboratory of Birth Defects and Related Diseases of Women and Children, Ministry of Education, West China Second University Hospital, Sichuan University, Chengdu, China

**Keywords:** Gut microbiota translocation, kidney microbiota, cell-wall-deficient bacteria, L-form bacteria, sIgA-coated bacteria, IgA protease, bacterial translocation, hypertension, dietary intervention

## Abstract

Gut microbiota dysbiosis promotes metabolic syndromes (e.g., hypertension); however, the patterns that drive hypertensive pathology and could be targeted for therapeutic intervention are unclear. We hypothesized that gut microbes might translocate to the kidney to trigger hypertension. We aimed to uncover their method of colonization, and thereby how to maintain blood pressure homeostasis. Using combined approaches based on fluorescence in situ hybridization (FISH) and immunofluorescence staining, electron microscopy analysis, bacterial cultures, species identification, and RNA-sequencing-based meta-transcriptomics, we first demonstrated the presence of bacteria within the kidney of spontaneously hypertensive rats (SHRs) and its normotensive counterpart, Wistar-Kyoto rats (WKYs), and patients with hypertension. Translocated renal bacteria were coated with secretory IgA (sIgA) or remained dormant in the L-form. *Klebsiella pneumoniae* (*K.pn*) was identified in the kidneys of germ-free (GF) mice following intestinal transplantation, which suggested an influx of gut bacteria into the kidneys. Renal bacterial taxa and their function are associated with hypertension. Hypertensive hosts showed increased richness in the pathobionts of their kidneys, which were partly derived from the gastrointestinal tract. We also demonstrated the indispensable role of bacterial IgA proteases in the translocation of live microbes. Furthermore, Tartary buckwheat dietary intervention reduced blood pressure and modulated the core renal flora-host ecosystem to near-normal states. Taken together, the unique patterns of viable and dormant bacteria in the kidney provide insight into the pathogenesis of non-communicable chronic diseases and cardiometabolic diseases (e.g., hypertension), and may lead to potential novel microbiota-targeted dietary therapies.

## Introduction

Hypertension is a dominant manageable cardiometabolic factor in non-communicable diseases and metabolic syndromes, with substantial public health burdens affecting approximately 30% of the adult population worldwide and contributing to approximately 19% of global deaths.^1,2^ As important endocrine and metabolic organs, the kidneys are severely affected by hypertensive damage^3,4^ and play a pivotal role in hypertension development and persistence.^5^ Because it is regulated by multiple factors, the relationship between the kidneys and hypertension remains unclear.

Many studies have evaluated the host-microbiota crosstalk in patients with hypertension, including in animals. Patients with diverse hypertension and pre-hypertension exhibit perturbed gut microbiota compared to healthy controls.^6^ However, renin-angiotensin system-related hypertension cannot be induced in germ-free (GF) animal models.^7^ Fecal microbiota transplantation from patients with hypertension into GF animals can elevate blood pressure (BP).^8^ Modulating the microbiota via probiotics or prebiotics ameliorates elevated BP levels and hypertensive damage to organs.^9,10^ A perturbed intestinal microbiota contributes to BP and renal function homeostasis.^11^ In addition to the microorganisms that normally inhabit barrier tissues, such as the gastrointestinal tract, bacterial signals have been observed in host organs and tissues that were previously considered sterile, such as the placenta,^12^ brain,^13^ and adipose tissues associated with proinflammatory cytokines.^14^ Bacterial loads were also detected in diverse human tumors, with bacteria occurring as intracellular L-form mimics,^15^ a cell wall-deficient state resistant to antibiotics and osmotic pressure, which can conceal them from host immune attacks.^16^ Additionally, IgA-coated bacteria, the first-line defense against immune exclusion,^17^ are associated with extraintestinal autoimmune disorders.^18^ Thus, bacteria with disease-causing potential that inhabit human internal organs influence the development of hypertension, although bacterial signals owing to environmental contamination cannot be ruled out during detection.

Gut microbiota have adapted to coexist in commensal or symbiotic relationships with mammals and systemically affect kidney function.^19,20^ Because the gut-vascular barrier controls the systemic dissemination of bacteria, gut bacteria cannot generally colonize the liver, kidney, or other internal organs in healthy individuals.^21^ However, bacteria colonize the liver^22^ and kidneys^22,23^ of healthy fish. Intestinal barrier dysfunction appears in spontaneously hypertensive rats (SHRs), a genetically predisposed model of hypertension derived from Wistar-Kyoto rats (WKYs), even in the pre-hypertensive juvenile state.^24^ Additionally, the majority of microorganisms detected in shock patients who developed infections were common bacteria that normally reside in the intestinal tracts, suggesting translocation of host bacteria in post-stroke infections.^25^ Therefore, gut microbiota could possibly colonize other organs in the body, which is a strong rationale to better understand hypertensive kidney microbiota. In addition to factors such as antibiotics, host genetics, and environment, which influence the composition of commensal and pathogenic microorganisms,^26^ diet can also quickly and repeatedly alter the microbiome of humans^27^ and mice.^28^ Therefore, we hypothesized that the translocation of live bacteria into the kidneys is associated with hypertension. We used strict experimental processing to eliminate contaminants and used fluorescence in situ hybridization, immunofluorescence staining, electron microscopy, bacterial transplantation, bacterial cultivation, and RNA-seq-based meta-transcriptomic profiling to confirm the results. We investigated the status, taxa, and functions of bacteria that could potentially inhabit organs or tissues using specified concentrations of L-forms and IgA-coated shapes. Moreover, we assessed the interactions between renal bacteria and colonized compartments within the host and their responses to dietary intervention, along with BP modulation.

## Results

### Bacterial detection in kidneys of hypertensive and normotensive rats at different weeks of age

To avoid exogenous bacterial contamination from environmental exposure, the specimens were aseptically transferred to a sterile biosafety cabinet, and the kidneys were dissected and fixed under sterile conditions. Sample collection and detection were performed under sterile conditions.^29^ The detailed experimental methods for renal tissue dissection and determination are shown in Fig. S1, similar to that in a previous study.^30^ Hand sterilization of the operators handling the tissue during dissection and processing was applied to control contamination from the operator; environment control was achieved by sterilizing the work surfaces such as hood, laminar flow, and work bench etc.; PBS and fixing buffer used at each step of tissue processing were prepared with sterile water and kept sterile. Operative procedures under sterile conditions, as well as sterile reagents, enable us to exclude the exogenous contamination caused by the environment and solvents. Tissue fixation solutions further prevent contamination with exogenous bacteria. Additionally, for bacterial culture, each procedure strictly followed established standards.^31^

**Figure 1.**
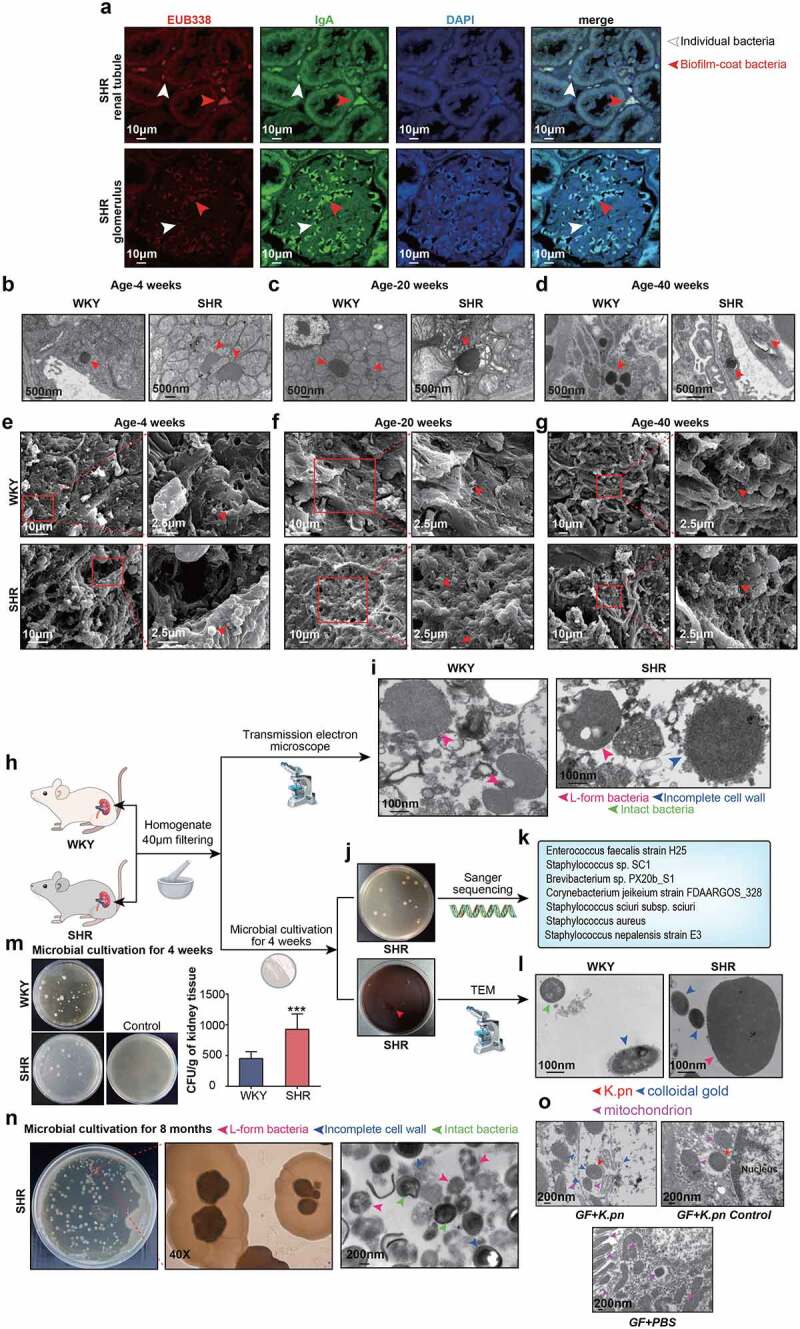
Viable bacteria within renal tissues. a, FISH probes targeting bacteria (EUB338) and IgA in paraffin sections of SHR renal tubules or glomeruli. Images show bacteria, IgA, and DAPI in red, green, and blue, respectively. Arrows indicate positive staining. Scale bars indicate size. n = 15/group. b–d, TEM of renal tissues from SHRs and WKYs shows the presence of bacteria within the cytoplasm of interstitial cells. Arrows indicate bacteria. n = 15/group. e–g, SEM of bacterial structures within the kidneys. The boxed region was selected for high magnification. Arrows indicate bacteria. n = 15/group. h, Kidneys were aseptically collected, homogenized, and subjected to TEM and *in vitro* culturing. i, TEM of bacteria in the renal homogenate. Arrows indicate bacteria with L-forms or incomplete cell walls. n = 15/group. j, Agar plates after 4 weeks of cultivation with the SHR kidney homogenate. k, Cultured bacteria were subjected to Sanger sequencing. Identified bacteria are listed. n = 15/group. l, TEM of renal bacteria cultured from SHRs and WKYs. Arrows indicate bacterial morphology. n = 15/group. m, Quantification of cultured bacteria from renal tissues of SHRs and WKYs. CFU, colony-forming units. Data are presented as the mean ± standard deviation. n = 6/group. ***p < 0.001. n, Microscopic images and SEM of bacteria are shown. Left: microbial cultivation on semisolid TSA for 8 months; middle: L-form “fried eggs” colonies under light microscope; right: SEM image. n = 15. o, Images for immune electron microscopy targeting *K.pn* in the kidneys of GF mice (n = 16) receiving *K.pn* gavage. Both samples (up left and up right) were from the renal tissues of GF recipient mice inoculated with *K.pn*. The “*K.pn* control” is a negative control of immunoelectron microscopy antibody against *K.pn*. Upper left image is from immunoelectron microscopy observations performed with *K.pn* antibody, and upper right is without, where no positive staining of colloidal gold detected, but bacterial structure was still labeled. Down, GF mice inoculated with sterile PBS buffer as naïve control also subjected to immunoelectron microscopy with their renal tissues (n = 19). Arrows indicate colloidal gold, mitochondrion, and *K.pn.*

**Figure 2.**
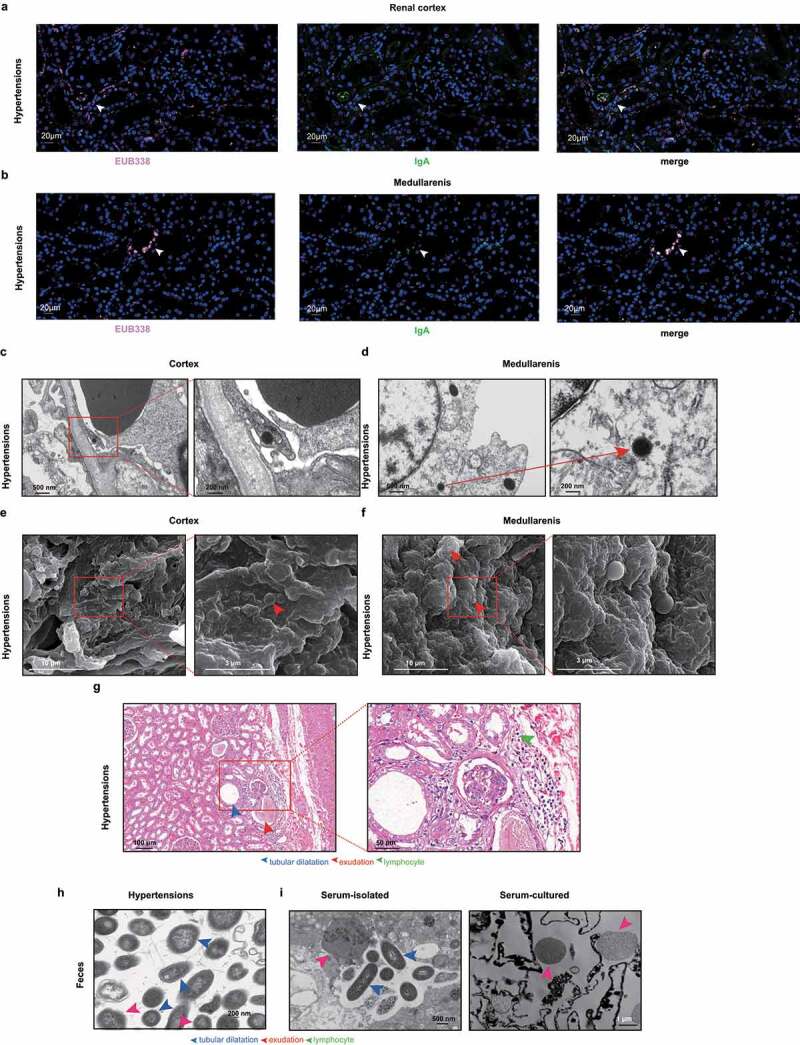
Morphological characteristics of bacteria from kidneys, feces, and serum samples of hypertensive patients. a, b, FISH images of bacteria (EUB338) and IgA in paraffin-embedded sections of the renal cortex 20× magnification (a) and medulla at 10× magnification (b) from hypertensive patients. Bacteria, IgA, and DAPI are in red, green, and blue, respectively. Arrows indicate bacteria and IgA-positive staining. n = 6. c, d, TEM analysis of the renal cortex (c) and medulla (d) from hypertensive patients indicates bacterial morphology inside the cytoplasm. The box region is magnified. Arrows indicate bacterial morphology. n = 6. e, f, Representative SEM images of the bacterial structures in the renal cortex (e) and medulla (f). The box region is magnified. Arrows indicate bacterial morphology. n = 6. g, Representative H&E staining of the renal tissue. n = 6. h, TEM of the fecal bacteria from hypertensive patients. Arrows indicate bacterial morphology. Scale bars are 200 nm. n = 6. i, Representative TEM images of bacterial morphology in isolated and cultured serum samples from hypertensive patients. Arrows indicate L-form bacteria and bacteria with incomplete cell walls. Scale bars indicate size. n = 6.

Fluorescence in situ hybridization (FISH) was performed using a universal probe (EUB338) combined with immunofluorescence to visualize bacteria in renal specimens from WKY and hypertensive animal models. Bacteria colocalized individually or concurrently with the distribution of secretory IgA (sIgA) as a biofilm-like morphology within renal tubules and in the glomeruli of SHRs and WKYs (Figure 1a, S2a, and S3a). In contrast, aliquots of the same samples were tested in parallel with probe non-Eub338 (complementary to Eub338 to control nonspecific binding) and without anti-sIgA antibody (to control sIgA nonspecific staining), which showed no nonspecific binding signals (Fig. S3b). Due to frequent clusters of bacteria-forming biofilms, quantifying the renal bacteria in the glomerulus was difficult; therefore, we quantified the bacteria within renal tubules, as fewer biofilms were observed in the clumps. Bacterial cells were substantially more abundant in SHRs than in WKYs (Fig. S4a). The colocalization of bacteria and sIgA was confirmed in renal specimens from hypertensive patients using FISH (Figure 2a,b). Intriguingly, sIgA-co-localized bacteria were also detected in SHR intestines (Fig. S5a) and further visualized using transmission electron microscopy (TEM) (Figs. S5b–d), in accordance with recent studies revealing that IgA-coated bacteria can invade the intestinal barrier^17,18^ and induce extraintestinal diseases by reducing virulence and increasing the invasive propensity.^32^ Although reported as a first-line firewall against “immune exclusion,^17^” sIgA may also aid in the pathogenicity of coated bacteria, potentially allowing for bacterial translocation and accumulation within the extraintestinal organs.

To further characterize and identify the renal bacteria, renal specimens were subjected to TEM and scanning electron microscopy (SEM). Cell wall-impaired bacterial structures were captured within the cytoplasm of interstitial cells in the renal tubules and glomeruli of SHRs and WKYs (Figures 1b–d). The bacterial cell walls observed were vague, thin, or completely deficient. Bacterial shape and size were highly variable and quite disparate with the common bacteria, raising the possibility of the cell wall-deficient bacteria known as the L-form. SEM provided a clearer visualization of coccoid structures (Figures 1e–g). At 4-weeks-old, when SHRs exhibited comparable blood pressure levels to WKYs, bacterial structures could be captured within the kidney. Therefore, renal bacteria were detectable before blood pressure elevation in SHRs, and thus were impossible to be the consequence of blood pressure changes. Similar bacterial morphology inside the cytoplasm was confirmed with low-grade inflammation in the renal cortex and medulla of patients with hypertension (Figure 2c–g). L-form-mimicking bacteria were also observed in the feces and sera of hypertensive animals and patients, indicating probable translocation of intestinal bacteria into the kidneys (Figure 2h,i and Figure S6a,b).

### Viable L-form bacteria within kidney

The bacteria observed using FISH, TEM, and SEM analyses were evaluated as nonviable upon arrival via passive bystanders or viable inhabitants in the kidney. Therefore, we attempted to isolate viable bacteria from the kidneys through cultivation. TEM revealed that bacteria in the renal homogenate exhibited disrupted cell walls (Figure 1h,i), which excluded the possibility of exogenous contamination, as almost all bacteria from the environment displayed intact cell walls. Using methods described previously,^31^ we performed bacterial cultivation with renal tissue lysates, and viable bacterial isolates were cultivated from the renal tissues of the SHRs and WKYs (Figure 1j–m). Generally, bacterial colonies are visible at 48–72 h during bacterial culture. Interestingly, we failed to detect bacteria within the first 3 days of bacterial culture of renal tissue lysates; it took at least 7 days when tiny bacterial colonies finally emerged. The extremely slow growth rate observed was in accordance with the characteristics of L-form bacteria. Our findings, based on bacterial culture and electron microscopy, indicated L-form bacteria within the renal tissue. Significantly more colonies were observed after microbial cultivation of kidney tissue from SHRs (Figure 1m). Considering obligate anaerobes and unculturable bacteria, it was difficult to accurately reflect the bacterial composition with the cultures. To provide evidence for the presence of viable bacteria, we performed Sanger sequencing on bacterial cultures from the kidney tissue of SHR as a representative. The taxa of the recovered isolates were identified as *Staphylococcus aureus* (*S.au*) by Sanger sequencing (Figure 1k), which did not accurately reflect the complete bacterial composition within the kidney tissue. Environmental bacterial contamination was random. Sanger sequencing (Figure 1k) identified specific bacteria that could not be randomly brought from the environment. Therefore, we ruled out exogenous bacterial contamination in the renal tissues.

The presence of *S. au* was further confirmed with *S. au*-specific FISH probes and colocalized with sIgA (Fig. S7a). Visualization of isolated bacterial colonies after culturing for 4 weeks indicated cell wall disruption as a typical L-form morphology (Figure 1l). After a prolonged 8-month period of culturing and subculturing, which allowed the “fried eggs” L-form colonies to grow, the bacteria were approximately spherical, lacked cell walls, and showed vague boundaries (Figure 1n and Figure S7b). L-form bacteria grew when they returned to the normal structure with the bacterial cell wall, and TEM images in Figure 1n showed bacteria with impaired, incomplete, or intact cell walls, indicating the progress of L-form bacterial recovery.

Due to their incomplete cell walls, L-forms are avirulent for effective immune responses; therefore, we speculated that they could exist in the kidneys without activating an immune attack and coexist with the host prior to inducing pathogenicity.^16,33^ L-form-shaped bacteria were also detected in the sera of both hypertensive rats and patients (Figure S6b and Figure 2i), demonstrating their potential to consistently survive in extraintestinal sites and translocate via circulation. To further confirm these bacterial structures, we applied immunogold staining specific to the strains within the kidneys. *K.pn* is an opportunistic pathogen that usually colonizes the lungs and intestines, causing diseases. Recently, phages targeting *Kpn* were found to protect against inflammatory bowel disease.^34^ The gut bacteria *K. pn* are highly abundant in patients with hypertension.^8^ Additionally, *K. pn*-induced damage to intestinal epithelial barrier integrity and enhanced intestinal permeability^35^ might allow it to leak into the extraintestinal sites. Thus, we used *K. pn* as a representative species to investigate the translocated bacteria in the kidneys. Upon oral transplantation of *K. pneumoniae* into gnotobiotic mice, positive *K. pn*eumoniae staining within the renal tissues of recipient mice was validated by immunoelectron microscopy (Figure 1o), which also indicated a possible influx of gut *K. pneumoniae* to the kidneys in gnotobiotic mice. *K.pn* elicited much higher systolic blood pressure in GF-recipient mice than in untreated controls.^36^ We suspect that the renal bacteria may be relevant to pyelonephritis or cystitis with exogenous pathogens deriving from the bladder and urinary tracts of these animals. Relatively uniform globular bacteria with low biomass inside the cytoplasm of the renal interstitial cells were considered endogenous bacteria, whereas exogenous bacteria differed randomly in abundance and morphology and could not reach the cytoplasm. Furthermore, globular bacteria in the glomerulus were excluded from transurethral infections, which would lead to the accumulation of flora, mainly in the renal pelvis. Within the kidneys of specific-pathogen-free animals, such as SHRs and WKYs, we could not rule out the possibility of bacteria derived from the bladder or ureters, as the intestinal tract might not be a unique source of bacteria.

### Bacterial IgA protease activation promotes bacteria translocation

**Figure 3.**
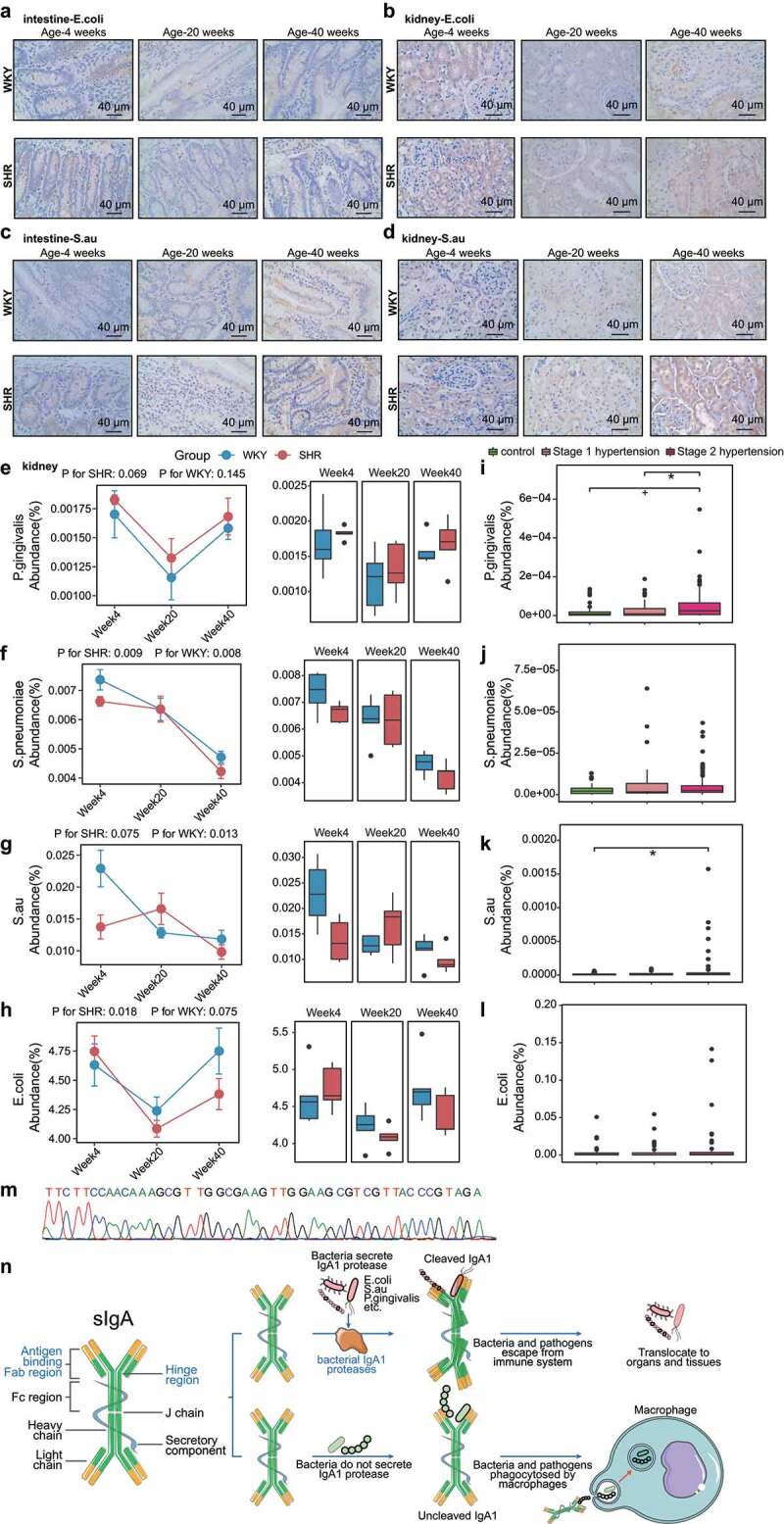
Bacteria with potential to secrete IgA1 protease within SHR and WKY renal tissues. a–d, Colonization and distribution of *E. coli* and *S.au* in the ileum segment of intestines and kidneys of SHRs and WKYs were determined by staining with specific antibodies targeting *E. coli* or *S.au*. Scale bars are 40 μm. n = 15/group. e–h, Quantitative abundances of *P. gingivalis, S. pneumoniae, S.au*, and *E. coli* within SHR and WKY renal tissues. n = 5/group. i–l, Relative abundances of *P. gingivalis, S. pneumoniae, S.au*, and *E. coli* in stool samples from healthy controls and patients with Stage 1 or Stage 2 hypertension. *p < 0.05, +p < 0.01, Wilcoxon rank-sum test. m, Renal tissues from SHRs were amplified by RT-PCR using primer pairs specific for the prtP-hydrolyzing IgA and IgG from *P. gingivalis*, and the cDNA fragment was confirmed by Sanger sequencing. n, sIgA cleaved by specific proteases derived from *E. coli, S.au* and *P. gingivalis* failed to deliver bacteria into macrophages but allowed bacteria to translocate to organs and tissues. n = 41 for control, n = 56 for Stage 1 hypertension, n = 99 for Stage 2 hypertension.

The sIgA coating restricts the pathogenic potential of targeted microorganisms.^37^ sIgA is vital for limiting cavity adhesion and colonization by microorganisms, but can be cleaved by specific proteases derived from bacteria such as *Escherichia coli* (*E. coli), S. aureus*, and *Porphyromonas gingivalis (P. gingivalis)*.^38^ Interestingly, we detected *E. coli* and *S. aureus* in both the kidneys and intestines of animals with hypertension (Figure 3a–d) and observed the profiles of *E. coli, S. aureus, S. pneumoniae*, and *P. gingivalis* within the SHR kidneys (Figure 3e–h). These bacteria, with the potential to secrete IgA protease, were detected in the gut microbiomes of patients with hypertension (Figure 3i–l), and the *P. gingivalis*-derived IgA protease gene amplified by PCR was sequenced and confirmed in the SHR renal tissues (Figure 3m) at the mRNA level. Thus, macrophages cannot identify IgA-coated strains without a hinge region, and the immune reaction is mitigated. Bacterial IgA protease is key in deleting parts of the host IgA and avoiding macrophage detection, ultimately enabling IgA-coated bacteria to pass through the cavity, reach the circulatory system, and adhere to the host organs (Figure 3n).

### Bacterial profiling and host transcriptome signatures in kidney

**Figure 4.**
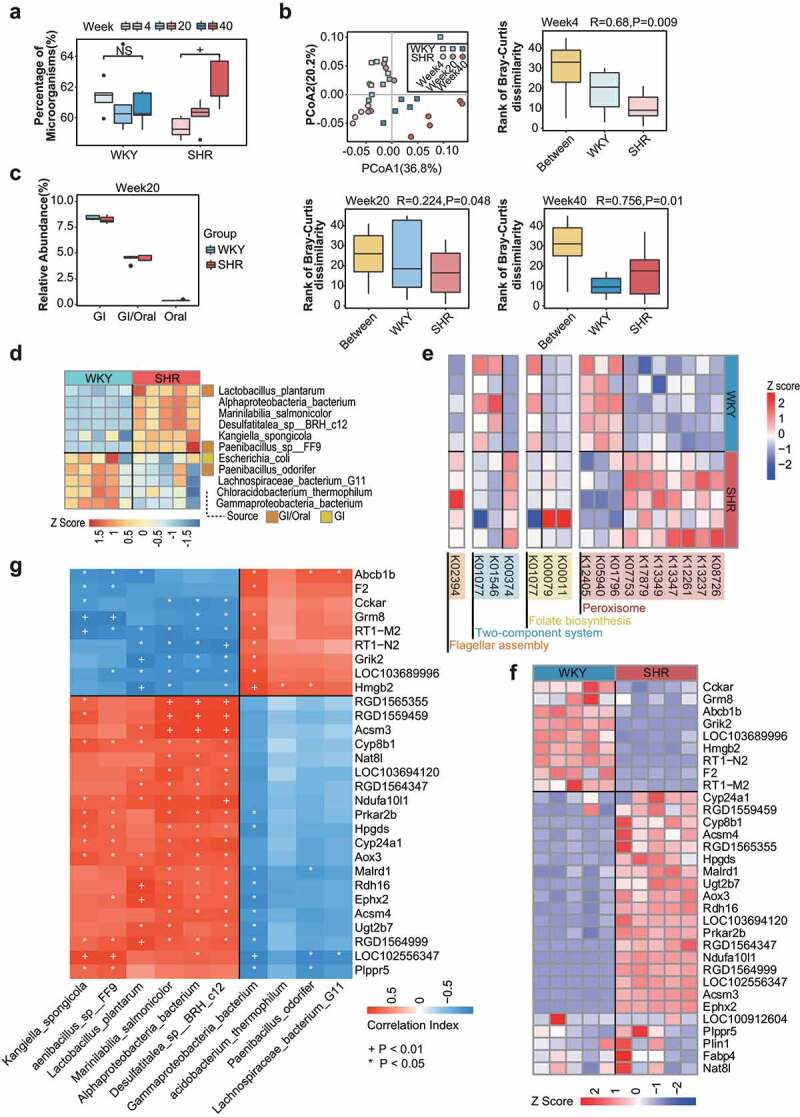
Kidneys contain a diverse and dynamic microbiota in healthy and hypertensive animals but the microbial profiles differ. a, Percentage of microorganismal genes in all genes detected. n = 5/group. b, PCoA and ANOSIM analyses of SHRs and WKYs based on species profiles. n = 5/group. c, Percentage of renal microorganisms originating from the gut or oral cavity in SHRs and WKYs at 20 weeks. n = 5/group. d, Bacteria discriminating SHRs from WKYs by LEfSe (LDA score >2.5). Microbial origins are indicated in colored squares. n = 5/group. e, Heatmap of significantly different KOs between SHRs and WKYs at 20 weeks, as determined by KEGG pathway analysis. These include peroxisomes, folate biosynthesis, quorum sensing, and a two-component system. n = 5/group. f, Heatmap of annotated host renal genes, which differ between SHRs and WKYs at 20 weeks. Gene expression levels are displayed by z-scores. g, Association of the genes from f and bacteria from d. GI, gastrointestinal. n = 5/group.

Next, we assessed whether these adaptive bacteria selectively penetrate the host barrier and survive in kidneys predisposed to hypertension. The taxa and functions of the microorganisms inhabiting the kidneys were analyzed via RNA-seq-based meta-transcriptomics to reveal the potential relationship between host renal genes and microorganisms. Bacterial reads were identified in the renal tissues from both SHRs and WKYs, suggesting the presence of bacteria within the kidneys (Figure 4a–f). Quantification of renal bacterial gene proportions in the kidneys revealed significant increases in SHRs along with persistent hypertension (Figure 4a and Table S1). Principal coordinate analysis (PCoA) based on the Bray-Curtis distance demonstrated that the bacterial community structures of the SHRs were distinct from those of WKYs at different ages, notably in the persistent hypertensive status (Figure 4b). Compositional analysis indicated that *Proteobacteria, Actinobacteria*, and *Firmicutes* were the most dominant phyla in both SHRs and WKYs (Fig. S8a); These phyla are also the predominant gut bacterial phyla in humans.^39^ Interestingly, more than 7.5% of renal bacteria (~55% of bacteria with known origins) were derived from gastrointestinal tract-specific flora (Figure 4c and Figure S9a and Table S2), which is consistent with our observation of the bacterial translocation of *K. pn* from the gastrointestinal tract (Figure 1o).

We further analyzed the differential renal taxa between the SHRs and WKYs. Renal species, such as *Alphaproteobacteria*, which are mainly characterized by intracellular persistence, distinguished SHRs from WKYs (Figure 4d and Fig. S9b and c, and Table S3). Among these, several bacteria originated from the gut (Figure 4d). Functional analysis of the renal microbiome was performed using the Kyoto Encyclopedia of Genes and Genomes (KEGG) database. Specific KEGG pathways differed between SHRs and WKYs, involving membrane-bound peroxisomes, folate biosynthesis, and the two-component system (Figure 4e and Table S4), which represented the potential for biofilm formation, resistance to phagocytosis, local environmental adaption, and bacterial growth.^40^ These features suggest that the environment in SHR kidneys may select for bacteria with long-term persistence potential, resulting in bacterial pathobiont accumulation in hypertensive hosts.

Enrichment analysis was performed on differentially expressed renal genes from the RNA-seq data to evaluate host features in response to the renal microbiota. Several host genes were distinct between the SHRs and WKYs (Figure 4f and Figure S10a–c, and Table S5). The key discriminatory genes correlated with the differential bacteria obtained (Figure 4f,g and Table S6). Specifically, cardiometabolic disease-associated genes, such as arachidonic acid metabolism-related cytochrome P450 family 8 subfamily B member 1 (*CYP8B1*), were enriched in SHRs. Additionally, genes in the SHRs were depleted relative to antigen processing and presentation (e.g., *RT1-M2*) (Fig. S10a–c, and Table S7). Both SHRs and WKYs were controlled under the same environment and normal chow diet, and the distinct bacterial community structures observed between WKY and SHR were considered to be elicited by their inherent genetic differences. Thus, local configurations within the host kidneys may adapt to microbes and potentially contribute to diseases.

### Dietary intervention restores core renal microbiota to lower BP

**Figure 5.**
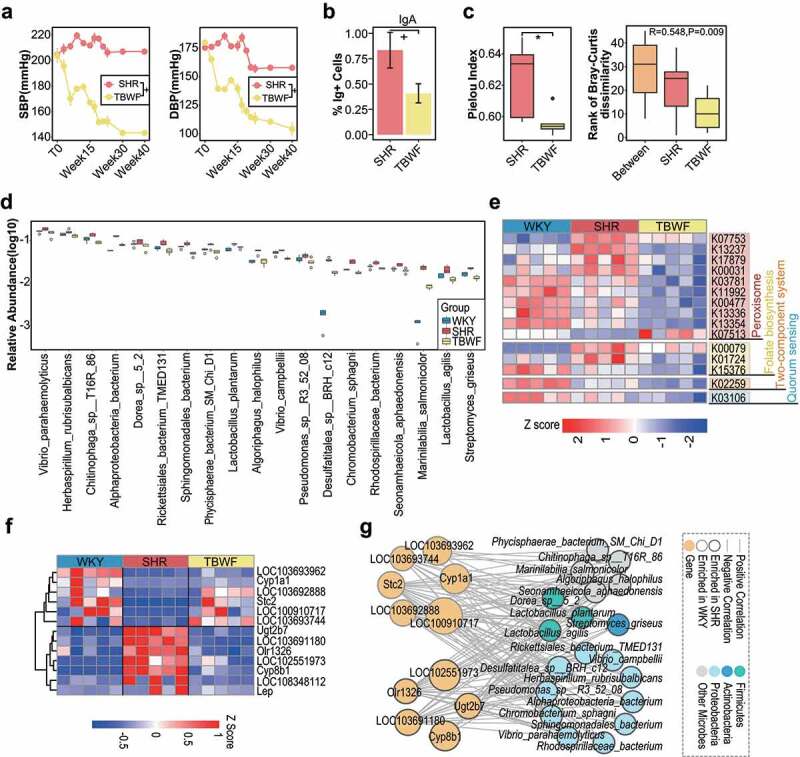
Renal microbial signatures were partly restored in SHRs after TBW intervention. a, Time-course changes of systolic BP (left) and diastolic BP (right) in SHRs under TBWF intervention (TBWF group, green-yellow, n = 5) and SHR controls (red, n = 5) at 40-week were measured by noninvasive tail-cuff. +p < 0.01, Wilcoxon rank-sum test. b, Percentage of IgA+ cells in TBWF group and SHR controls based on fluorescence-activated cell sorting. +p < 0.01, *p < 0.05, Wilcoxon rank-sum test. n = 5/group. c, alpha-diversity of Pielou index and ANOSIM analysis (Bray-Curtis dissimilarity) of TBWF group and SHR controls based on renal microbial profiles at species level. *p < 0.05; NS, not significant. n = 5/group. d, Relative abundance of the top 19 renal bacterial species distinct in SHRs from WKYs was restored via TBWF intervention as analyzed by LEfSe, LDA score >2.0. n = 5/group. e, Heatmap shows 15 KOs distinguishing controls from TBWF-treated SHRs. n = 5/group. f, based on host transcriptional profiles, the heatmap shows 13 renal genes that distinguished SHRs from WKYs and were restored by TBWF intervention. n = 5/group. g, Correlation network (red line, positive correlation; blue line, negative correlation) of 19 bacterial species from d and 13 host genes (yellow) from f. Colors of species represent bacterial phyla. Colored borders of nodes indicate enrichment in SHRs (red) and WKYs (blue).

Tartary buckwheat (TBW) is a traditional staple food for the Chinese Yi ethnic population, which is reported to have a low incidence of hypertension,^41^ and is used to prevent hypertension in Chinese populations.^42^ Derived from TBW, 2-HOBA can return the blood pressure of mice with hypertension almost to normal,^43^ and was validated as safe as a nutritional supplement in clinical trials (ClinicalTrials.gov numbers: NCT03555682, NCT03176940, and NCT03554096). Recently, TBW has attracted attention because of its high-quality nutritional composition, usability with other grains, and gluten-free properties.^44^ With the rapid accumulation of studies on microbiota, the probiotic/prebiotic properties of TBW and its effects on microbiota are noteworthy. Hence, studies on the effects of TBW on the host microbiome are new and promising in animal models. We used a TBWF containing TBW (50%) and *Chenopodium quinoa* (50%) to serve as a candidate diet for modulating the gut microbiota^45,46^ and host BP homeostasis^47,48^ in SHRs. We aimed to explore the similarity between the microbial composition of SHR after TBWF intervention and that of WKY controls. Renal microbial taxa, functions, and differentially expressed host genes were restored and BP was modulated after TBWF intervention (Figure 5a–g). As expected, both BP levels and proportions of IgA^+^ cells in SHRs were restored following TBWF administration (Figure 5a,b). The renal bacterial α-diversity of the Pielou indices decreased, and β-diversity, as indicated by the Bray-Curtis distance, showed pronounced separation between the SHRs and TBWF-treated SHRs (Figure 5c). To explore the core renal species that interact with BP attenuation, we examined the bacterial composition of WKYs, SHRs, and TBWF-treated SHRs. We integrated the data of kidney microbiome sequencing from WKYs and SHRs, and microbiome changes with TBWF dietary intervention, to find common threads that link these results together. Among the 85 differential species between SHRs and WKYs, 23 renal bacterial species were significantly restored in the SHRs via TBWF intervention (Figure 5d and Fig. S11a and b). Bacterial functions associated with peroxisomes, folate biosynthesis, quorum sensing, and two-component systems in SHRs were also modified by TBWF dietary intervention (Figure 5e and Figure S11c). Expression of host genes such as the cardiometabolic disease-associated, G protein-coupled receptor-related *Olr1326* gene, and the aryl hydrocarbon receptor pathway of antibacterial defense-associated *CYP1A1* was reversed after the intervention (Figure 5f). TBWF-targeted flora and genes were significantly correlated based on the bacterium-to-gene networks (Figure 5g). The discrepancy between SHR and SHR+TBWF was believed to be caused by diet rather than environment, blood pressure reduction, and genetics. As the blood pressure of SHRs would not be attenuated without TBWF intervention, we ruled out the notion that reduced blood pressure directly leads to microbial shifts. The TBWF diet affected renal bacteria by altering the gut microbiome, and not by improving the blood pressure of SHRs. Dietary intervention with TBWF has been suggested to influence the composition of the gut microbiota, resulting in fewer bacteria translocated into the renal tissues, reconstructing the core microbiota within the kidney, and ultimately lowering blood pressure. Adaptive bacteria may translocate to and inhabit host organs and interact with the host transcriptome, thereby modulating BP. However, the causative mechanisms of the renal microbiota in hypertension remain unknown.

## Discussion

Mounting evidence indicates that bacteria within sterile organs are potentially physiological and pathology-promoting.^14,29,30,49–52^ However, whether the intestinal microbiota can access the liver, kidney, or other internal organs in healthy individuals remains controversial,^12,30^ especially because of technical challenges. Different factors, such as exposure of experimental samples to the environment, reagents, PCR, sequencing processes, and methodology instruments can lead to false-positive microbial signals. Hence, during our experiments, all renal samples were collected, fixed under sterile conditions, and analyzed using RNA-seq. RNA-based meta-transcriptomics, rather than DNA-based shotgun metagenomics, was performed to identify viable microbiotas and their transcriptional profiles. The confounding presence of damaged, non-viable bacteria, or contaminant DNA was excluded. In addition, sequencing resolution of species and strain levels were achieved, although limitations on sequencing were particularly involved in the loss of microbial gene expression and relative bacterial abundances provided by gene transcription, which may thus partially deviate from the actual bacterial abundances. Intracellular bacteria were further confirmed in the kidney tissues via TEM, which eliminated possible contamination from the external environment to the greatest possible extent. Using combined approaches, we demonstrated for the first time the presence of viable bacteria within hypertensive and normotensive kidneys, especially in spontaneously hypertensive rats with pre-hypertension (normotension) at 4-weeks-old, and illustrated their alterations, which are associated with hypertension. These results indicate that dysbiosis of the kidney microbiota is the cause, rather than the result, of hypertension.

Notably, sIgA-coated bacteria exist within the renal tissues of both animals and patients with hypertension, even in biofilm forms. sIgA is generally the first-line defense of the host immune system against infection by eliminating invasive pathogens and maintaining mucosal commensals.^53^ Significantly, the selectively outgrowing IgA-coated microbes genetically and functionally resemble a microbial pathotype with reduced virulence factors but increased adherent or invasive propensity and replication potential in macrophages.^32^ sIgA-coated bacteria were also detected in SHR and WKY intestines. Thus, sIgA coating assists in bacterial colonization or persistence in the organs via antibody-enhanced biofilm formation. We also observed that pathogenic bacteria such as *E. coli*,^54^ *S.au*,^55^ and *P. gingivalis*^38^ within the kidneys of hypertensive animals could secrete IgA1 proteases at mucosal sites of infection. Unlike sIgA-coated bacteria normally phagocytosed by macrophages, bacteria secreting IgA1 proteases, which were detected in patients with hypertension in our study, could damage the structure and functions of human IgA1, thereby abolishing host IgA defenses by cleaving specific peptides in the IgA1 hinge region.^56^ Moreover, the IgA structure contributes to antigenic heterogeneity, as demonstrated by IgA1 proteases during infection and the cleavage specificity of IgA1 proteases for human IgA1.^57^ Therefore, IgA1 proteases have been implicated as core persistence factors that induce bacterial infection and colonization.

Recently, selective IgA deficiency in SHR has been proven to be a host factor associated with gut microbiota dysbiosis.^58^ Serum IgA, which can eliminate a large number of antigens, including bacteria and viruses, and maintain the stability of the internal environment without eliciting inflammation, was determined in SHRs with disordered intestinal flora.^58^ Here, we mainly focused on the crucial role of secretory IgA in the translocation of gut microbiota to extraintestinal organs, as IgA is known to selectively bind to specific bacterial species and exert important functions during bacterial colonization in the intestinal tract.^18,59^ Immune responses to serum IgA and secretory IgA are disparate. It was speculated that both renal translocation of secretory IgA-encapsulated bacteria digested by IgA protease found in the present study, and selective deficiency of serum IgA in SHRs reported previously,^58^ produce similar consequences during hypertension, but the underlying mechanisms might be quite different. The potential correlations between these findings and hypertension pathogenesis warrant further investigation.

The L-forms act in a special cell-wall-deficient bacterial state with attenuated virulence to adapt to the environment, showing increased resistance to wall-active antibiotics such as β-lactams, and the potential to switch back to the pathogenic walled state.^33^ The innate immune effector lysozyme^16^ coupled with antibiotic abuse induces bacterial transformation into the L-form, and hypertonic conditions often concurrent with hypertension, such as high salt and hyperglycemia,^60^ can facilitate the persistent survival of L-form bacteria. Here, we first observed “dormant” L-forms with limited onsite inflammation in the kidneys (organs originally thought to be sterile) of hypertensive hosts via TEM, SEM, bacterial culturing, and staining, indicating that the L-form state is a potential element of bacterial persistence within the kidneys. Thus, the gut microbiota may bypass the host immunological filter using the host defense mechanism of IgA or undergo adaptive bacterial transformations, such as the L-form, to inhabit the kidneys.

Recent investigations have revealed that bacteria translocate to establish niches in organs that were once thought to be sterile, including adipose tissues, the liver, spleen, pancreas, and brain.^14,49–52,61^ To our knowledge, this study provides the first evidence of viable bacteria in the kidneys of humans and animal models of hypertension, including juveniles. Microbes such as *Alphaproteobacteria*, characterized by intracellular persistence, were determined via meta-transcriptomic profiling to inhabit the kidneys of hypertensive animals. An increased proportion of microorganisms and accumulated pathobionts such as *K. pneumoniae* are observed along with persistent hypertension, which share the capacities required for biofilm formation and persistence,^62^ and are correlated with high-BP phenotypes. Interestingly, animal hypertensive models exhibit increased gut wall permeability prior to the clinical phenotype, resulting in pathological intestinal changes in adult rodents.^24^ A gut-vascular barrier appears to prevent microbial components from entering systemic circulation.^21^ Here, source-tracking analysis showed that microbes residing in the kidneys could translocate from the gastrointestinal tract. Both *K. pn* inoculation into GF animals and immunogold staining of renal specimens confirmed that bacteria can translocate from the gastrointestinal tract into the kidneys.

IgA-producing cells specifically binding gut-encountered antigens can reach the circulation and extraintestinal organs, such as atherosclerotic plaques and the brain.^63,64^ Investigators have confirmed that some plasma cells in the central nervous system are mobilized from the gastrointestinal tract. For instance, IgA-promoting commensal microbes drive gut-derived IgA-producing plasma cells and elicit protection against neuroinflammation and resistance to experimental autoimmune encephalomyelitis.^65^ Meanwhile, suppressed IgA-binding fecal bacteria detected during acute relapse of multiple sclerosis patients also supports contention in humans.^65^ Furthermore, IgA, as a key regulator at the mucosal interface, responds to intestinal bacterial surface antigens in the gut, binds to specific fecal microbiota, and exerts immunostimulatory capacity in activating central nervous system autoimmune diseases such as multiple sclerosis.^66^ Specific gut bacteria have been suggested to be potent IgA inducers implicated in neuroinflammation and multiple sclerosis pathogenesis.^66^ Therefore, IgA-coated gut bacteria might access the brain and are implicated in hypertension.^67^ In the present study, secretory IgA co-localized with *K. pn* within the renal tissue of GF-recipient mice, which raises the possibility that IgA-coated bacteria might also translocate into the brain and affect the central nervous system. Further studies directly uncovering the influence of IgA-coated bacteria such as *K. pn* on the gut-brain axis would provide a better explanation for hypertension pathogenesis.

Intervention with renal bacteria via TBWF revealed that changes in the kidney microbiota can modulate host BP, and antibiotics were excluded for the following reasons: i) widespread prevalence of antibiotics in neonatal and childhood populations can disrupt microbial homeostasis, eliminate beneficial bacteria,^68^ and be a risk factor for metabolic syndromes;^69^ ii) antibiotics may induce cell-wall-impaired L-forms,^16,33^ which are the core bacterial morphology in hypertensive host kidneys; and iii) intracellular microbes in the kidneys, such as *Alphaproteobacteria* bacteria, may become antibiotic-resistant. Conversely, dietary formulas such as the dietary approaches to stop hypertension (DASH) or Mediterranean diets, which are marked by the consumption of more complex carbohydrates and high-fiber ingredients, including fruits, vegetables, and whole grains, are beneficial in managing cardiometabolic disease.^70^ The microbial ecosystem can ferment these ingredients and produce metabolites, thus improving gut barrier function and host homeostasis.^71^ High-fiber diets and acetate supplements have also shown BP-lowering and heart/kidney-protecting effects in DOCA-salt hypertensive models.^10^ Non-pharmacological modulation of diets would be more natural, safer, and cost-effective in real-world settings. Thus, early life dietary interventions may help recover the microbial ecosystem and maintain host BP homeostasis.

However, the DASH diet was not employed in this study, as it is not applicable to Eastern populations.^70^ Instead, we used a diet of TBW derived from that followed by the Yi community in Sichuan Province, southwest China; this population has a metabolic syndrome prevalence of only 2.4%.^41^ In 1937, Morse et al. reported much lower BP levels and extremely low incidences of hypertension in the Chinese Yi population.^72^ The Yi people mainly consume TBW in their diet, which is rich in quercetin, 2-hydroxybenzylamine,^43^ flavonoids, and omega-3 fatty acids, and can normalize BP and protect against cardiovascular injuries.^73^ A recent cross-sectional study in northern Germany also revealed an inverse association between flavonoid-rich food consumption and systolic BP levels, with microbial factors explaining over 15% of this association.^74^ Thus, we used TBWF dietary supplements as interventions in hypertensive rats, which concurrently modulated BP levels and the renal microbe-host ecosystem, and made them more similar to those of the healthy controls. Further investigations of TBWF dietary interventions in hypertensive populations are warranted.

Our study has several limitations. First, we only examined samples from SHRs with essential hypertension and found IgA-coated bacteria or L-forms, which were confirmed in biopsy samples from patients with essential hypertension. In a rat model of secondary hypertension, deoxycorticosterone acetate (DOCA)-salt hypertensive rats and Dahl salt-sensitive (SS) rats (SSR) remain to be determined. SSR is a typical model of secondary hypertension caused by the interaction between a high-salt diet (environmental factor) and genetic factors. A high-salt diet inevitably leads to high osmolarity in the gastrointestinal tract, which is conducive to the translocation and colonization of L-forms. Similarly, essential hypertension is also characterized by a genetic component that interacts with environmental risk factors including diet. Possibly, genetic predisposition alone may not play as great a role as is currently believed. The kidney microbiome, could be a factor independent of host heredity, and is transmitted from parents. Second, the data based on live bacteria detected in the kidney from 4-week-old SHRs with normal BP and TBWF intervention reconstructing hypertensive renal flora to lower blood pressure suggest that kidney microbiota dysbiosis is the cause rather than the result of hypertension. However, we could not confirm the bacterial species that promote the occurrence and development of hypertension.

In this study, we first elucidated that viable bacteria colonized the renal tissue from hypertension and normotension. Most notably, these bacteria exhibited L-forms or IgA-coated forms, likely with attenuated virulence with limited potential for an immune attack but improved persistence and the potential to switch to a pathogenic state. Our analysis of renal bacterial taxa and their functions revealed that some gut microbes can easily penetrate or bypass host defenses to reach available niches with less pathogenic potential. Microbiome influence many physiological and pathological processes, and diet is a key factor that substantially affects the composition and function of these microbial communities. Dietary intervention with TBWF, a diet traditionally followed by the Chinese Yi population, helps maintain host BP homeostasis, protects the gut barrier, and modulates BP and the renal microbial ecosystem analogous to those of healthy controls. The host-immune system crosstalk among translocated bacteria and its role in the pathogenesis of renal hypertension, and the role of TBWF dietary interventions in microbial ecosystem recovery and host homeostasis in hypertensive populations, deserve further study.

## Materials and methods

### Animals and ethics

SH and WKY rats were purchased from Beijing Vital River Laboratory Animal Technology Co. Ltd. (Beijing, China) and housed individually under specific pathogen-free conditions. Kidney and blood samples from SHRs and WKYs were aseptically collected in a biosafety cabinet at 4, 20, and 40 weeks. GF C57BL/6 L mice were obtained from the Shanghai Institutes for Biological Sciences (SLAC Inc., Shanghai, China) and housed under a 12-h light–dark cycle in gnotobiotic facilities. All mice were fed sterile food and water *ad libitum*, and bacterial contamination was monitored by periodic examination of the stool samples. The sample sizes were determined based on prior experience with similar trials and are indicated in the figure legends. SHR and GF C57BL/6 L mice were randomly assigned to different groups, as needed. The Animal Care and Use Committee of West China Hospital, Sichuan University, approved the research protocols, and the animal experiments were performed according to the guidelines of the Animal Ethics Committee.

SHRs were bred from normotensive WKY rats, which are generally used to control SHRs in animal models of hypertension. In this study, we found live bacteria within the kidney in both developing SHRs and WKY rats. Therefore, other rat strains would be inappropriate as positive or negative controls for the SHRs.

### Clinical samples

In this study, 6 hypertensive renal biopsies were performed. The demographics are shown in Table S8. Renal biopsy specimens were used for histopathological analysis, together with blood and fecal samples, and were collected from hypertensive patients admitted to the Fuwai Hospital. The diagnosis of hypertension was based on the 2010 Chinese guidelines for the management of hypertension.^75^ Individuals who had received antibiotic or probiotic therapy within the last 8 weeks were excluded. Ethical approval was obtained from the institutional ethics board of the Fuwai Hospital. Data collection and analysis of cases were required by the National Health Commission of the People’s Republic of China to be part of a continuing public health outbreak investigation. All the participants provided informed consent.

Kidney biopsies were performed in accordance with previous reports.^76^ A nephrologist performed percutaneous kidney biopsy using an automated spring-loaded biopsy needle (16-gauge, Bard Magnum, Bard Biopsy Systems, USA) with ultrasound guidance. The renal biopsies were immediately collected in a sterile operating room, the fresh tissue was divided and placed in 4% buffered formaldehyde and 3% glutaraldehyde + 1% paraformaldehyde in 0.1 M phosphate buffer. The formaldehyde-fixed tissue was embedded in paraffin, sectioned, and stained with hematoxylin-eosin (H&E). The tissue for TEM (3% glutaraldehyde + 1% paraformaldehyde in a 0.1 M phosphate buffer) was embedded in epoxy resin, cut into 60 nm thin sections, and observed under a transmission electron microscope (JEOL, Beijing, China).

Blood samples collected on the day of the biopsy were centrifuged for 30 min, and plasma/serum was placed in plastic vials. The vials were stored in at −80°C freezer pending further analysis. Fresh stool samples were collected using sterile manure collectors. The middle portion was collected using a sterile fecal sampler. Approximately 0.2 g of each stool sample was weighed in a sterile centrifuge tube and aliquoted into a new 1.5-ml Eppendorf tube. Each sample was transferred for storage at −80°C for 20 min until use.

### Combination of FISH and immunofluorescence staining

Bacteria within the kidneys were identified in paraformaldehyde-fixed tissue sections. The ileum segment of intestinal tissues was examined as previously reported.^77^ As pathological changes have been detected in the small intestine of SHRs,^24^ the ileum of the intestine was found to be suitable for assessing intestinal changes. Paraffin-embedded tissues were deparaffinized and hybridized to a universal bacterial probe (EUB338:5′-GCTGCCTCCCGTAGGAGT-3′), control probe (non-EUB838:5′-ACTCCTACGGGAGGCAGC-3′), or a probe specific for *S. aureus* with the sequence 5′-GAAGCAAGCTTCTCGTCCG-3′. All probes were labeled with the Cy5 fluorophore and obtained from Sangon Biotech Co., Ltd. (Shanghai, China). Hybridization was performed overnight at 56°C, followed by washing and counterstaining with the nuclear dye,2-(4-Amidinophenyl)-6-indolecarbamidine dihydrochloride (DAPI). FISH and immunofluorescence staining were performed on slides first stained with a bacterial FISH probe. Slides were washed thrice with phosphate-buffered saline (PBS), blocked with 1% bovine serum albumin for 30 min, and incubated with anti-sIgA antibody (Abcam, ab17921) in a humidified chamber at 4°C overnight. The slides were incubated with fluorescent-conjugated secondary antibodies at room temperature for 30 min after washing thrice with PBS. The tissues were washed thrice with PBS and counterstained with DAPI.

### Hematoxylin and eosin (H&E) and immunostaining

The tissues were fixed in 4% paraformaldehyde (PFA) overnight at 4°C and washed thrice with PBS before storing in 70% ethanol. Tissues were routinely processed, paraffin-embedded, sectioned, and stained using Molecular Pathology Core at Sichuan University. Tissues were immune-stained using paraffin-embedded slides that were de-paraffinized, and antigen retrieval was performed by incubating the samples in citrate buffer (Sigma-Aldrich, St. Louis, MO, USA) heated in a microwave oven. The samples were subsequently incubated in blocking buffer (5% normal goat or mouse serum in PBS, depending on the species of primary antibody) for 30 min at room temperature and then incubated with one of the following primary antibodies: anti-IgA secretory component antibody (Abcam, ab212330); anti-*Staphylococcus aureus* (Abcam, ab20920). After washing thrice with PBS, the tissue samples were incubated with fluorescent-conjugated secondary antibody, followed by DAPI nuclear staining. Biotinylated secondary antibody was added, followed by staining with an avidin-biotin complex and counterstaining with hematoxylin.

### Imaging analysis

Bright-field and fluorescent images were acquired using an Optronics Microfire charge-coupled device camera on a Leica DM2000 upright compound microscope. Confocal imaging was performed using LSM 700 (403 oil-immersion objective lens; Carl Zeiss) or A1R (603/1.4 oil-immersion objective lens; Nikon). The images were analyzed using either ZEN software or FUJI Image J.

### Scanning electron microscopy (SEM)

For SEM analysis, SHR and WKY kidneys were collected under strictly sterile conditions (Supplementary material S1), fixed in 10% formalin for at least 24 h, and dehydrated in increasing concentrations of ethanol (30%, 50%, 70%, 80%, 90%, 95%, and 100%). Following dehydration, the samples were incubated in 97% 1,1,1,3,3,3-hexamethyldisilazane until dry. The samples were mounted on stubs using adhesive tape, metallized with gold, and subsequently analyzed using an FEI Inspect microscope (Thermo Fisher, Waltham, Massachusetts, USA) operating at 20 kV.

### Transmission electron microscopy (TEM)

TEM was performed as previously described by Donaldson.^59^ Briefly, a 1 cm portion of the renal cortex, renal medulla, and ileum segment of intestinal tissues from SHRs and WKY rats was excised under strictly sterile conditions (Supplementary material S1) and immediately fixed with an ice-cold solution of 3% glutaraldehyde, 1% paraformaldehyde, and 5% sucrose in 0.1 M sodium cacodylate trihydrate. Tissues were prefixed for 1 h at 4°C, then transferred to a petri dish containing 5% sucrose in 0.1 M cacodylate buffer. Tissues were cut into ~1-2 mm^3^ blocks with a scalpel and placed into brass planchettes prefilled with cacodylate buffer containing 10% Ficoll (70 kD, Sigma-Aldrich), which serves as an extracellular cryoprotectant. Excess buffer was removed using Whatman filter paper, and the sample was covered with a Type B brass planchette. Samples were ultra-rapidly frozen using an HPM-010 high-pressure freezing machine and then transferred immediately under liquid nitrogen to cryotubes (Nunc) containing a frozen solution of 2.5% osmium tetroxide and 0.05% uranyl acetate in acetone. The tubes were loaded into an AFS-2 freeze-substitution machine (Leica Microsystems) precooled to −100°C. Samples were processed at −90°C for 72 h, warmed over 12 hours to −20°C, held at that temperature for 6–10 hours, then warmed to 4°C for 1 h. The fixative was removed and the samples were dehydrated through an acetone series from 30%, 50%, 70%, 80%, 90%, 95%, and 100%, washed thrice with 100% acetone, and finally embedded in epoxy resin. Resin was polymerized at 60°C for 48 h. Ultrathin sections were cut using a diamond knife (Diatome) on an ultramicrotome (Leica) and stained with uranium acetate and lead citrate. Electron micrographs were recorded using a JEOL JEM-1400Flash electron microscope (JEOL, Beijing, China) operating at 80 kV.

In this study, renal intracellular bacteria were endogenous, not exogenous, because strict aseptic operations were performed to avoid environmental contamination, and exogenous bacteria could not enter the renal cell cytoplasm during the short experimental period. To identify bacteria in the cytoplasm of renal cells, sections were surveyed until a bacterium was recognized in the cytoplasm of renal cells. Bacterial cells were identified based on morphology, and more than five different fields were analyzed per sample. Approximately 1 mm of the renal cortex and medulla were scanned per rat (15 SHRs and WKY rats, respectively).

### Immunoelectron microscopy

For immunoelectron microscopy, the samples were fixed with 4% paraformaldehyde and 0.5% glutaraldehyde, dehydrated in acetone, and embedded in epoxy resin. Sections were cut and attached to gold or nickel grids and stained with anti-*K. pn* antibody (ab20947), followed by secondary antibodies conjugated to 15-nm gold beads, and then post-stained with uranyl acetate and lead citrate. The electron micrographs were recorded using a JEOL JEM-1400Flash electron microscope operating at 80 kV.

### Bacterial cultures and species identification

Bacteria were cultivated and the species were identified as described previously.^31^ Briefly, samples were aseptically collected, weighed, and immediately transferred to a Cell Strainer (BD Falcon, Cat 352350) for manual homogenization in 1 ml of pre-reduced (autoclaved, sterile filtered at 0.22 μm, and oxygen reduced with vacuum degasification) PBS + 0.1% l-cysteine (Sigma-Aldrich, 168149). Homogenates (100 μl per plate) were plated on Columbia blood agar (Guangdong Huankai Microbial Sci. & Tech. Co., Ltd.) and incubated anaerobically. For aerobic cultures, tissue homogenates were plated onto tryptone bile agar (TBA) (Guangdong Huankai Microbial Sci. & Tech. Co., Ltd.) and incubated aerobically. The plates were sealed with Parafilm and incubated upside-down in a bacteriological incubator. After 2 days (aerobic) or 5 days (anaerobic), the formation of bacterial colonies was observed every 2 days. Colony-forming units (CFUs) were quantified after a minimum 4-week culturing under constant temperature and humidity. The results were expressed as CFU/g of tissue.

The representative bacterial colonies grown on the agar plates were picked with sterile pipette tips and stored at −80°C until further analysis. The bacterial colonies were thawed at room temperature and resuspended in 6 μl sterile water to lyse the bacteria by heating them at 95°C for 10 min. The samples were subsequently centrifuged at 12,000 rpm for 10 min, and 2 μl supernatant was used as a template to amplify the 16S rRNA gene using universal 16S rRNA primers (27 F, 5′-AGTTTGATCMTGGCTCAG-3′ and 1492 R, 5′-GGTTACCTTGTTACGACTT-3′) using PCR under the following reaction conditions: 94°C for 4 min, followed by 30 cycles at 94°C for 45s, 55°C for 45s, 72°C for 1 min, and finally 72°C for 10 min. The amplification product (10 μL) was incubated with 2 μL of ExoSAP-IT (Thermo Fisher, 78200.200.Ul) at 37°C for 15 min, followed by a second incubation at 80°C for 15 min. The amplicons were sequenced using capillary sequencing (Shanghai Sangon Biotech Co., Ltd.), and the resulting sequences were compared using BLAST against the 16S ribosomal RNA sequence database to identify the species.

### *K.pn* culture and transplantation

Previous studies^8,78^ showed enrichment of *K.pn* in the gut of individuals with hypertension compared with normotensive individuals, and *K.pn* was also rich in SHR kidneys in this study. Therefore, *K. pneumoniae* were transplanted to confirm that intestinal bacteria can translocate to the kidney. *K. pn* (ATCCBAA-1144) was purchased from MicroBioLogics, Inc. (https://www.microbiologics.com). *K.pn* was cultured according to the manufacturer’s instructions, washed twice with sterile PBS, and resuspended in sterile saline at a concentration of 10^9^ per mL for oral gavage. GF C57BL/6 L mice were raised in Trexler-type flexible film isolators at the Institute of Laboratory Animal Sciences, Chinese Academy of Medical Sciences, and Comparative Medicine Center, Peking Union Medical College. GF mice were randomly divided into two groups receiving *Kpn* and PBS. All mice were housed at 22 ± 1°C, with a relative humidity of 50 ± 1%, and a 12/12-hr light/dark cycle, and fed an autoclaved standard chow diet *ad libitum*. GF mice were administered via oral gavage every two days at 100 μL of ~10^9^ CFU/mL *K.pn*. After transplantation, recipient mice were housed in gnotobiotic facilities and fed sterile food and water for 8 weeks. All foods used for the gnotobiotic experiments were either autoclaved or irradiated to ensure sterility. All animal studies and euthanasia of mice were performed in compliance with the regulations and guidelines of the Peking Union Medical College’s Institutional Animal Care and conducted according to the AAALAC and IACUC guidelines.

### Transcript analysis of the IgA protease gene prtP of *P. gingivalis*

To confirm the presence of the bacterial IgA protease gene at the mRNA level in hypertensive kidneys, the following primers were used: prtP, forward 5′- CTACGGGTAACGACGCTTCCAAC −3′, reverse 5′- CCTGAGCACGAGTACCACGAATG −3′ spanning a 108-bp region of the *P. gingivalis*porphypain (prtP) gene transcript (RefSeq: U42210.1); and GAPDH (housekeeping gene), forward 5′-GGTGAAGGTCGGAGTCAACGGA-3′ and 5′-GAGGGATCTCGCTCCTGGAAGA-3′ (Sangon Biotech). Total RNA was extracted from renal tissue using TRIzol reagent (Invitrogen), and RNA quality was assessed using an Agilent 2100 BioAnalyzer before reverse transcription into cDNA with Maxima H Minus Mastermix following the manufacturer’s instructions (Thermo Fisher Scientific). RNA (1 μg) was used to synthesize single-stranded cDNA using a high-capacity cDNA reverse transcription kit (catalog number #4368814), according to the manufacturer’s instructions. This fragment was amplified using reverse transcription PCR (RT-PCR). The PCR products were run on an agarose gel to visualize amplicon expression, and then purified using the QIAquick PCR purification kit, followed by product quantification and Sanger sequencing.

### Relative abundances of *P. gingivalis*, *Streptococcus pneumoniae*, *S.au*, and *E. coli* in patients

In our previous study, we sequenced fecal DNA samples via metagenomic analyses of 41 healthy controls, 56 patients with stage 1 hypertension, and 99 patients with stage 2 hypertension, and determined the abundance of the annotated taxa.^8^ The relative abundances of *P. gingivalis, S. pneumoniae, S. aureus*, and *E. coli* were determined in the gut microbiome of these participants.

### sIgA^+^ cell sorting

IgA^+^ plasma cells obtained from SHRs and TBWF-treated SHRs were stained with primary antibodies specific for sIgA (ab 17921, Abcam, 1:100 dilution) for 30 min at 4°C, followed by washing and incubation with anti- mouse antibody conjugated to Alexa Fluor 488 (A-21206, Invitrogen, 1:100 dilution) for 30 min at 4°C, and then collected on a FACSCanto II (BD Biosciences). Data were captured using the FACSDiva software (v. 9.0) and analyzed using the FlowJo software (v. 10.7.1).

### TBWF dietary intervention

Spontaneous hypertension in rats is very similar to essential hypertension in man.^79^ Hypertension in SHRs develops initially without any obvious organic lesions and mainly with hemodynamic alterations due to increased peripheral vascular resistance. Antihypertensive agents, which are useful for essential hypertension, are also effective in SHR.^79^ Therefore, it is an ideal animal model to study human essential hypertension and screen hypertensive drugs. Two diets were used in the current study: a standard laboratory chow diet (Lab Diet 5012) and a TBWF diet containing TBW (50%) and *Chenopodium quinoa* (50%). Eleven-week-old SHRs were acclimatized to a standard laboratory chow diet for a week in a specific-pathogen-free facility, and were randomly divided into two groups: the standard chow diet group and dietary intervention group. The diet in the experimental group was then changed to a TBWF diet, and the control groups were fed a normal chow diet. All diets were provided *ad libitum* for 28 weeks, unless otherwise noted. Blood pressure was measured using a noninvasive tail-cuff system (SoftronBP-98A; Softron, Tokyo, Japan). All SHRs experiments were replicated at least twice with the same TBWF.

### RNA isolation

RNA isolation was performed as previously described by Donaldson.^59^ Kidneys were freshly harvested under strictly sterile conditions on a workbench (Supplementary material S1), and immediately lysed by bead-beating in a mixture of 500 μl buffer (0.2 M NaCl and 20 mM EDTA), 210 μl 20% SDS, 500 μl phenol, chloroform, and isoamyl alcohol (Ambion AM9720). The aqueous phase was separated by centrifugation and transferred to a new tube for second extraction with phenol, chloroform, and isoamyl alcohol. Subsequently, 50 μl of 3 M sodium acetate and 500 μl of cold ethanol were mixed into the aqueous fraction and placed on ice for 20 min. RNA was pelleted, washed once with cold 70% ethanol, and then resuspended in 100 μl water. RNA was further purified using the Qiagen RNeasy Mini Kit, according to the manufacturer’s instructions. DNA was removed using Turbo DNase (Ambion AM2238) for one hour at 37°C before applying to a second Qiagen RNeasy column, including an on-column Qiagen RNase-free DNase digestion.

### RNA sequencing (RNA-Seq)

Renal tissues were obtained and total RNA was extracted using an RNeasy kit (Qiagen) with DNase. RNA degradation, contamination, and DNA contamination were monitored on 1.5% agarose gel. RNA concentration and purity were measured using the NanoDrop 2000 Spectrophotometer (Thermo Fisher Scientific, Wilmington, DE, USA). RNA integrity was assessed using the RNA Nano 6000 Assay Kit on the Agilent Bioanalyzer 2100 System (Agilent Technologies, CA, USA). RNA (1.5 μg per sample) was used as the input material for rRNA removal using the Ribo-Zero rRNA Removal Kit (Epicenter, Madison, WI, USA). Sequencing libraries were generated using the NEBNext^R^Ultra^TM^ Directional RNA Library Prep Kit for Illumina^R^ (NEB, USA) according to the manufacturer’s recommendations, and index codes were added to attribute sequences to each sample. The index-coded samples were clustered on the acBot Cluster Generation System using TruSeq PE Cluster Kitv3-cBot-HS (Illumina), according to the manufacturer’s instructions. After cluster generation, libraries were sequenced on an Illumina HiSeq platform, and paired-end reads were generated.

### RNA-seq analysis

After quality control using Trimmomatic, the CLC Genomics Workbench (version 12.0.3, CLC Bio, Aarhus, Denmark) was used to map the high-quality reads against the rat genome (Rnor6.0) and perform RNA-Seq statistical analyses. RNA-Seq analyses were performed using the rat genome using the following parameters: length fraction =  0.8, similarity fraction =  0.8, mismatch cost = 2, insertion cost = 3, and deletion cost = 3. The expression values were set as transcripts per million (TPM). A differential expression analysis test was used to compare gene expression levels and identify differentially expressed genes (DEGs) with a cutoff of absolute Log_2_ fold change (FC) values>2 and FDR p-values<0.05. Functional enrichment analyses of DEGs were performed using the cluster Profiler package (version 3.16.1) in R.

### Bacterial detection in RNA-seq data

Unmapped next-generation sequencing reads are typically ignored; however, they contain biologically relevant information.^80^ Algorithms (e.g., PathSeq^81^) were developed to perform computational subtraction of human reads, followed by alignment of residual reads to human reference genomes/transcriptomes and microbial reference genomes (including bacterial, viral, archaeal, and fungal sequences). These alignments resulted in the taxonomic classification of reads into bacterial, viral, archaeal, and fungal sequences in the RNA-seq data.^82^ Raw RNA-seq data were quality-filtered and cleaned using Trimmomatic (version 0.36).^83^ Following quality control, only sequences with perfect or near-perfect matches to the rat genome (Rnor6.0, alignment with SOAP2, version 2.21)^84^ were removed. In addition, SortMeRNA (Version 2.1b)^85^ was used to predict and remove small RNAs against SILVA (Version 119)^86^ and Rfam (Version 14)^87^ rRNA databases.

For taxonomic classification, the remaining reads were considered putative bacterial mRNA reads and were used to predict bacterial abundance using Kaiju (Version 1.7.3)^88^ with MEM mode against the ‘nr_euk’ dataset, which included protein sequences for bacteria, archaea, viruses, fungi, and microbial eukaryotes constructed from the NCBI nr database. Based on the species profiles, the linear discriminant analysis effect size (LEfSe) (Version 1.0)^89^ was used to identify significantly different species between groups. Following the taxonomic classification of non-rat DNA sequencing reads, the relative abundance value for each bacterial organism was calculated using reads mapped with ≥90% sequence identity and ≥90% query coverage. Classifications were performed at the domain, phylum, genus, and species levels, requiring unique alignments (i.e., reads with equivalent E-values to multiple taxa were removed from the analysis). The species-level relative abundance (RA) for each organism was calculated as follows: relative abundance of a given organism in a sample = (number of unique alignment positions across the genome × 1,000,000)/ (number of total aligned bacterial reads × bacterial genome size). The RA values were then normalized such that the total relative abundance for each sample added up to one (or 100% for percentage relative abundance).

For functional annotation, putative bacterial reads were assembled *de novo* into contigs using MEGAHIT (version 1.1.1-2-g02102e1)^90^ with default parameters. Genes were predicted from these scaffolds using MetaGeneMark (version 3.38),^91^ and the predicted proteins (minimum length:100 nucleotides) were clustered using CD-HIT (version 4.7).^92^ To determine gene abundance, the reads were realigned to the non-redundant gene catalog using SOAP2. Only genes with ≥2 mapped reads were deemed present in the sample. Gene abundance was calculated by counting the number of reads and normalizing them to gene length. KEGG orthologs (KOs) of the genes were annotated using Ghost KOALA (Version 2.2).^93^ KO abundance was calculated by summing the abundance of genes annotated to the same feature. Differential KO abundances were tested using the Wilcoxon rank-sum test in R (version 4.0.0). P-values were corrected for multiple testing using the Benjamini-Hochberg method.

### Statistical analysis

α-Diversity (within-sample diversity) was estimated based on the species profiles using R (version 4.0.0, package “vegan”). The Wilcoxon rank-sum test was used to compare differences in α-diversity between the groups. β-diversity (between-sample diversity) was estimated based on the species profiles by the Bray-Curtis distance using R (Version 4.0.0, package “vegan”). The difference in beta diversity among the groups was determined using ANOSIM analyses. Principal coordinate analysis (PCoA) was performed using the R package “vegan.” The R package “ggplot2” was used to visualize the results. The Wilcoxon rank-sum test was used to compare the differences between the groups. Taxonomic data on the NCBI taxonomy information of the reference genomes were obtained from the NCBI taxonomy database. Based on the phylogenic information and species profile, the source of the species was defined, and the proportions of different groups were calculated. CFU are represented as mean ± standard deviation. Statistical analyses were performed using independent t-tests in GraphPad Prism version 8.

## Supplementary Material

Supplemental Material

## Data Availability

The RNA-seq data of this study have been deposited in the European Nucleotide Archive (ENA) under the BioProject accession code PRJEB42560 [http://www.ebi.ac.uk/ena/data/view/PRJEB42560].
