# Supplementary material for: Kidney microbiota dysbiosis contributes to the development of hypertension
Source: Gut Microbes. 2022 Nov 12;14(1):2143220. doi: 10.1080/19490976.2022.2143220 (PMC9662196; doi:10.1080/19490976.2022.2143220)
Supplement: Supplemental Material [file KGMI_A_2143220_SM3919.zip › Supplementary Figure 1_11.pdf]

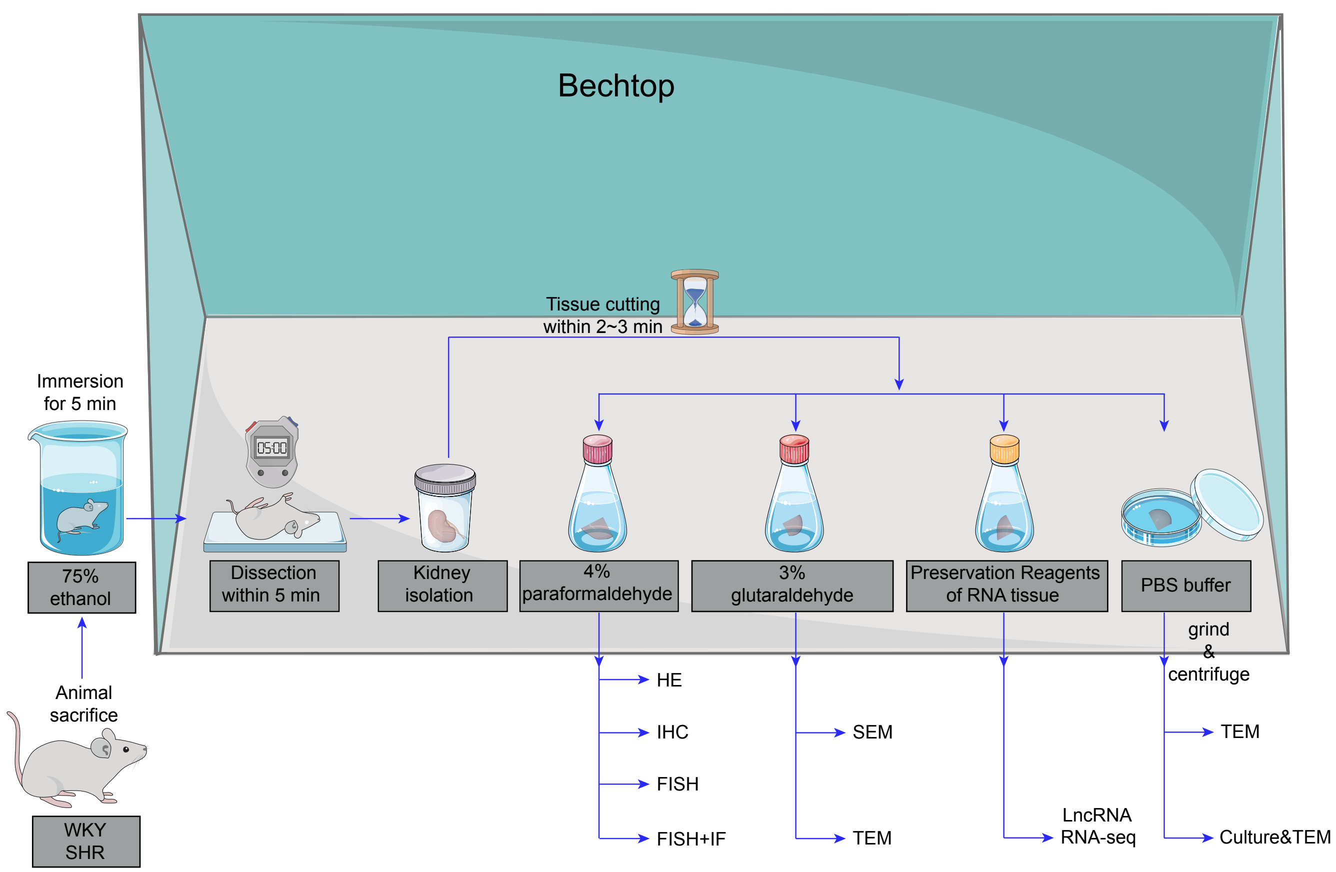

Bechtop
Tissue cutting
within 2~3 min
Immersion
for 5 min
05:00
75%
ethanol
Dissection
within 5 min
Kidney
isolation
4%
paraformaldehyde
3%
glutaraldehyde
Preservation Reagents
of RNA tissue
PBS buffer
grind
&
centrifuge
HE
IHC
FISH
FISH+IF
SEM
TEM
LncRNA
RNA-seq
TEM
Culture&TEM
Animal
sacrifice
WKY
SHR

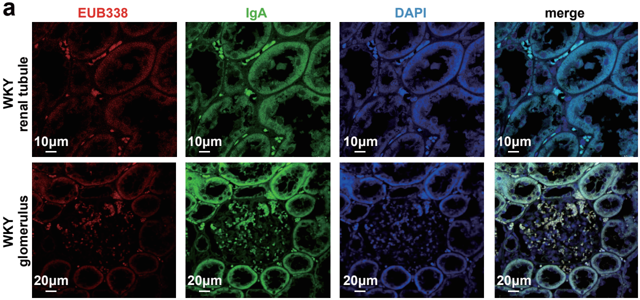
a
EUB338
IgA
DAPI
merge
WKY renal tubule
WKY glomerulus
10μm
20μm

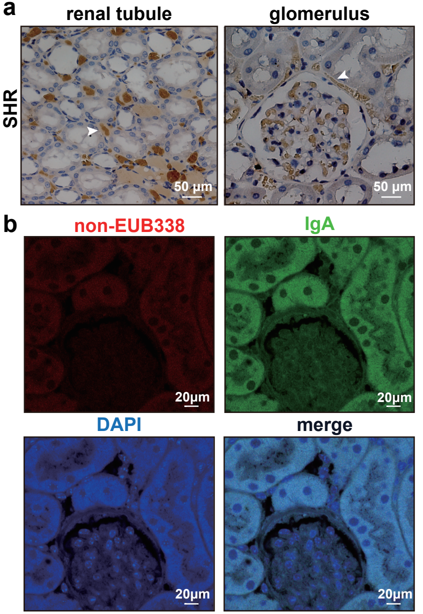
a
renal tubule
glomerulus
SHR
50 µm
50 µm
b
non-EUB338
IgA
20µm
20µm
DAPI
merge
20µm
20µm

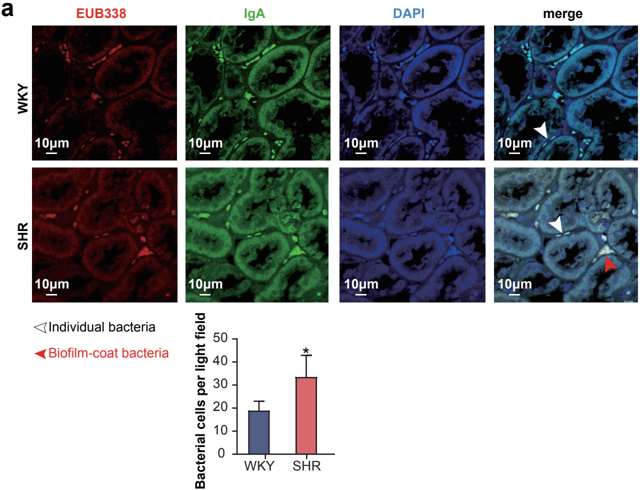

a
EUB338
IgA
DAPI
merge
WKY
SHR
10μm
Individual bacteria
Biofilm-coat bacteria
Bacterial cells per light field
50
40
30
20
10
0
*
WKY
SHR

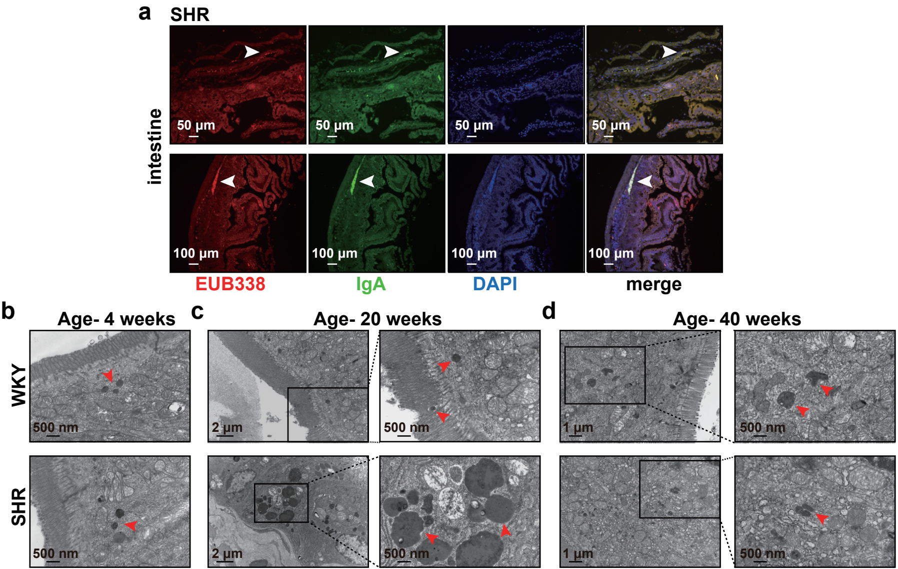

a
SHR
intestine
50 μm
100 μm
EUB338
IgA
DAPI
merge
b
Age- 4 weeks
WKY
SHR
500 nm
c
Age- 20 weeks
2 μm
500 nm
d
Age- 40 weeks
1 μm
500 nm

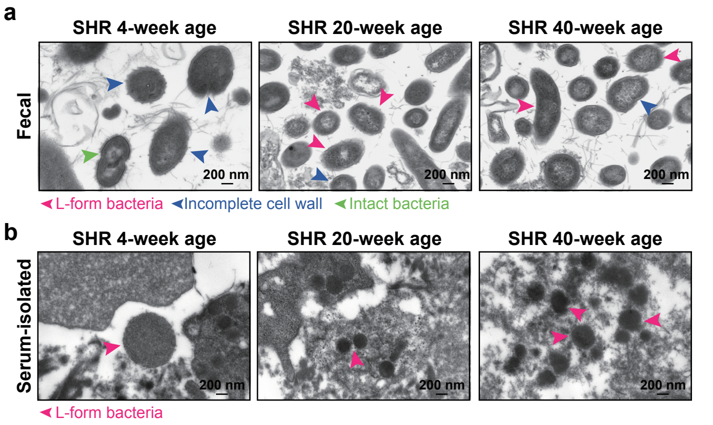
a
SHR 4-week age
SHR 20-week age
SHR 40-week age
Fecal
200 nm
200 nm
200 nm
L-form bacteria
Incomplete cell wall
Intact bacteria
b
SHR 4-week age
SHR 20-week age
SHR 40-week age
Serum-isolated
200 nm
200 nm
200 nm
L-form bacteria

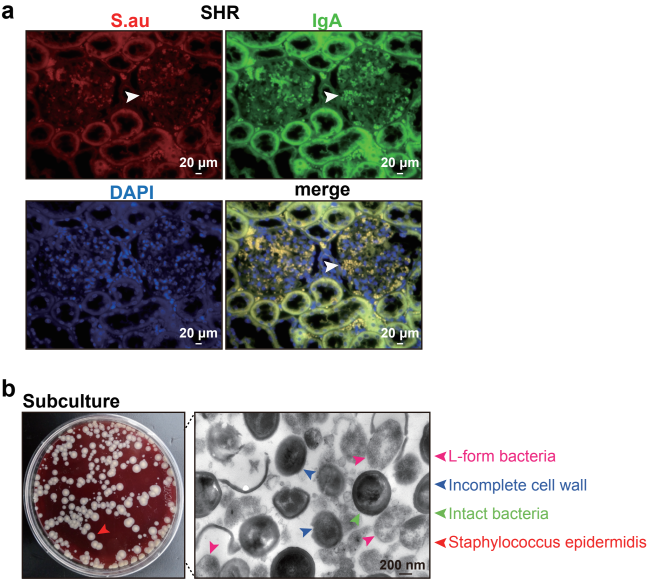
a
S.au
SHR
IgA
20 μm
20 μm
DAPI
merge
20 μm
20 μm
b
Subculture
200 nm
L-form bacteria
Incomplete cell wall
Intact bacteria
Staphylococcus epidermidis

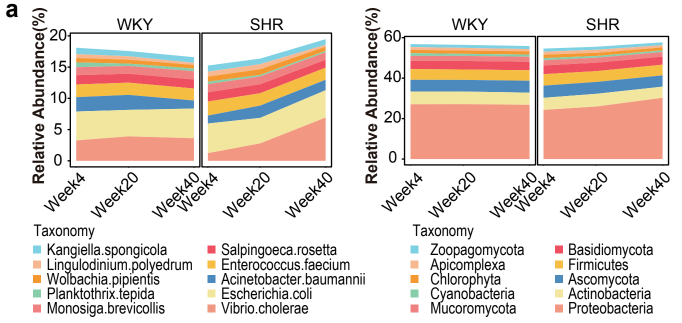

a
WKY
SHR
Relative Abundance(%)
20
15
10
5
0
Week4
Week20
Week40
Taxonomy
Kangiella.spongicola
Lingulodinium.polyedrum
Wolbachia.pipientis
Planktothrix.tepida
Monosiga.brevicollis
Salpingoeca.rosetta
Enterococcus.faecium
Acinetobacter.baumannii
Escherichia.coli
Vibrio.cholerae
60
40
Zoopagomycota
Apicomplexa
Chlorophyta
Cyanobacteria
Mucoromycota
Basidiomycota
Firmicutes
Ascomycota
Actinobacteria
Proteobacteria

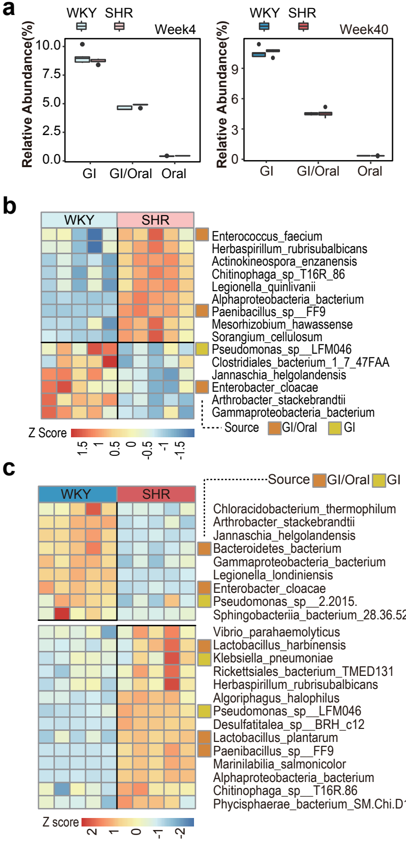

a
WKY
SHR
Week4
Relative Abundance(%)
10.0
7.5
5.0
2.5
0.0
GI
GI/Oral
Oral
WKY
SHR
Week40
Relative Abundance(%)
9
6
3
0
GI
GI/Oral
Oral
b
WKY
SHR
Enterococcus_faecium
Herbaspirillum_rubrisubalbicans
Actinokineospora_enzanensis
Chitinophaga_sp_T16R_86
Legionella_quinlivanii
Alphaproteobacteria_bacterium
Paenibacillus_sp__FF9
Mesorhizobium_hawassense
Sorangium_cellulosum
Pseudomonas_sp__LFM046
Clostridiales_bacterium_1_7_47FAA
Jannaschia_helgolandensis
Enterobacter_cloacae
Arthrobacter_stackebrandtii
Gammaproteobacteria_bacterium
Source
GI/Oral
GI
Z Score
1.5
1
0.5
0
-0.5
-1
-1.5
c
Source
GI/Oral
GI
WKY
SHR
Chloracidobacterium_thermophilum
Arthrobacter_stackebrandtii
Jannaschia_helgolandensis
Bacteroidetes_bacterium
Gammaproteobacteria_bacterium
Legionella_londiniensis
Enterobacter_cloacae
Pseudomonas_sp__2.2015.
Vibrio_parahaemolyticus
Lactobacillus_harbinensis
Klebsiella_pneumoniae
Rickettsiales_bacterium_TMED131
Herbaspirillum_rubrisubalbicans
Algoriphagus_halophilus
Pseudomonas_sp__LFM046
Desulfatitalea_sp__BRH_c12
Lactobacillus_plantarum
Paenibacillus_sp__FF9
Marinilabilia_salmonicolor
Alphaproteobacteria_bacterium
Chitinophaga_sp__T16R.86
Z score
2
1
0
-1
-2

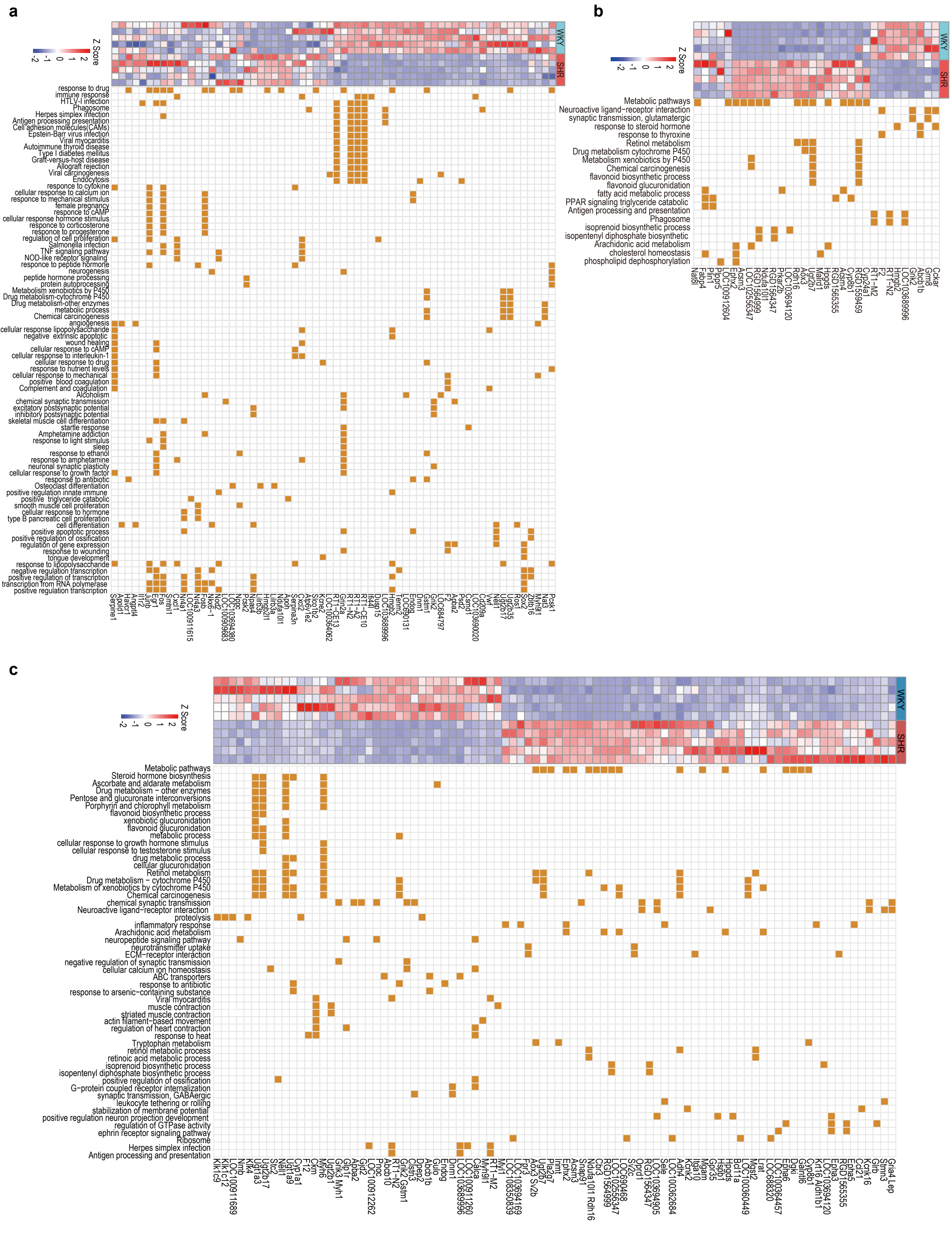
a
Z Score
2
1
0
-1
-2
WKY
SHR
response to drug
immune response
HTLV-I infection
Phagosome
Herpes simplex infection
Antigen processing presentation
Cell adhesion molecules(CAMs)
Epstein-Barr virus infection
Viral myocarditis
Autoimmune thyroid disease
Type I diabetes mellitus
Graft-versus-host disease
Allograft rejection
Viral carcinogenesis
Endocytosis
responce to cytokine
cellular response to calcium ion
responce to mechanical stimulus
female pregnancy
responce to cAMP
cellular response hormone stimulus
responce to corticosterone
responce to progesterone
regulation of cell proliferation
Salmonella infection
TNF signaling pathway
NOD-like receptor signaling
responce to peptide hormone
neurogenesis
peptide hormone processing
protein autoprocessing
Metabolism xenobiotics by P450
Drug metabolism-cytochrome P450
Drug metabolism-other enzymes
metabolic process
Chemical carcinogenesis
angiogenesis
cellular response lipopolysaccharide
negative extrinsic apoptotic
wound healing
cellular response to cAMP
cellular response to interleukin-1
cellular response to drug
response to nutrient levels
cellular response to mechanical
positive blood coagulation
Complement and coagulation
Alcoholism
chemical synaptic transmission
excitatory postsynaptic potential
inhibitory postsynaptic potential
skeletal muscle cell differentiation
startle response
Amphetamine addiction
response to light stimulus
sleep
response to ethanol
response to amphetamine
neuronal synaptic plasticity
cellular response to growth factor
response to antibiotic
Osteoclast differentiation
positive regulation innate immune
positive triglyceride catabolic
smooth muscle cell proliferation
cellular response to hormone
type B pancreatic cell proliferation
cell differentiation
positive apoptotic process
positive regulation of ossification
regulation of gene expression
response to wounding
tongue development
response to lipopolysaccharide
negative regulation transcription
positive regulation of transcription
transcription from RNA polymerase
positive regulation transcription
b
Z Score
2
1
0
-1
-2
WKY
SHR
Metabolic pathways
Neuroactive ligand-receptor interaction
synaptic transmission, glutamatergic
response to steroid hormone
response to thyroxine
Retinol metabolism
Drug metabolism cytochrome P450
Metabolism xenobiotics by P450
Chemical carcinogenesis
flavonoid biosynthetic process
flavonoid glucuronidation
fatty acid metabolic process
PPAR signaling triglyceride catabolic
Antigen processing and presentation
Phagosome
isoprenoid biosynthetic process
isopentenyl diphosphate biosynthetic
Arachidonic acid metabolism
cholesterol homeostasis
phospholipid dephosphorylation
c
Z Score
2
1
0
-1
-2
WKY
SHR
Metabolic pathways
Steroid hormone biosynthesis
Ascorbate and aldarate metabolism
Drug metabolism – other enzymes
Pentose and glucuronate interconversions
Porphyrin and chlorophyll metabolism
flavonoid biosynthetic process
xenobiotic glucuronidation
flavonoid glucuronidation
metabolic process
cellular response to growth hormone stimulus
cellular response to testosterone stimulus
drug metabolic process
cellular glucuronidation
Retinol metabolism
Drug metabolism – cytochrome P450
Metabolism of xenobiotics by cytochrome P450
Chemical carcinogenesis
chemical synaptic transmission
Neuroactive ligand–receptor interaction
proteolysis
inflammatory response
Arachidonic acid metabolism
neuropeptide signaling pathway
neurotransmitter uptake
ECM–receptor interaction
negative regulation of synaptic transmission
cellular calcium ion homeostasis
ABC transporters
response to antibiotic
response to arsenic–containing substance
Viral myocarditis
muscle contraction
striated muscle contraction
actin filament–based movement
regulation of heart contraction
response to heat
Tryptophan metabolism
retinol metabolic process
retinoic acid metabolic process
isoprenoid biosynthetic process
isopentenyl diphosphate biosynthetic process
positive regulation of ossification
G–protein coupled receptor internalization
synaptic transmission, GABAergic
leukocyte tethering or rolling
stabilization of membrane potential
positive regulation neuron projection development
regulation of GTPase activity
ephrin receptor signaling pathway
Ribosome
Herpes simplex infection
Antigen processing and presentation

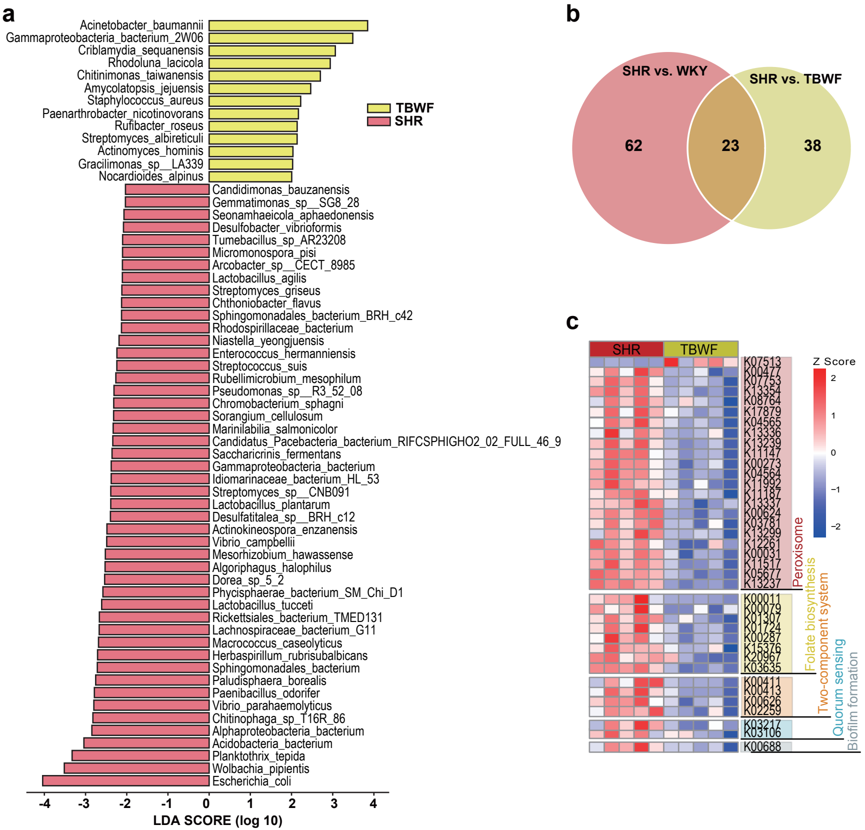

a
Acinetobacter_baumannii
Gammaproteobacteria_bacterium_2W06
Criblamydia_sequanensis
Rhodoluna_lacicola
Chitinimonas_taiwanensis
Amycolatopsis_jejuensis
Staphylococcus_aureus
Paenarthrobacter_nicotinovorans
Rufibacter_roseus
Streptomyces_albireticuli
Actinomyces_hominis
Gracilimonas_sp__LA339
Nocardioides_alpinus
Candidimonas_bauzanensis
Gemmatimonas_sp__SG8_28
Seonamhaeicola_aphaedonensis
Desulfobacter_vibrioformis
Tumebacillus_sp_AR23208
Micromonospora_pisi
Arcobacter_sp__CECT_8985
Lactobacillus_agilis
Streptomyces_griseus
Chthoniobacter_flavus
Sphingomonadales_bacterium_BRH_c42
Rhodospirillaceae_bacterium
Niastella_yeongjuensis
Enterococcus_hermanniensis
Streptococcus_suis
Rubellimicrobium_mesophilum
Pseudomonas_sp__R3_52_08
Chromobacterium_sphagni
Sorangium_cellulosum
Marinilabilia_salmonicolor
Candidatus_Pacebacteria_bacterium_RIFCSPHIGHO2_02_FULL_46_9
Saccharicrinis_fermentans
Gammaproteobacteria_bacterium
Idiomarinaceae_bacterium_HL_53
Streptomyces_sp__CNB091
Lactobacillus_plantarum
Desulfatitalea_sp__BRH_c12
Actinokineospora_enzanensis
Vibrio_campbellii
Mesorhizobium_hawassense
Algoriphagus_halophilus
Dorea_sp_5_2
Phycisphaerae_bacterium_SM_Chi_D1
Lactobacillus_tucceti
Rickettsiales_bacterium_TMED131
Lachnospiraceae_bacterium_G11
Macrococcus_caseolyticus
Herbaspirillum_rubrisubalbicans
Sphingomonadales_bacterium
Paludisphaera_borealis
Paenibacillus_odorifer
Vibrio_parahaemolyticus
Chitinophaga_sp_T16R_86
Alphaproteobacteria_bacterium
Acidobacteria_bacterium
Planktothrix_tepida
Wolbachia_pipientis
Escherichia_coli
TBWF
SHR
-4 -3 -2 -1 0 1 2 3 4
LDA SCORE (log 10)
b
SHR vs. WKY
SHR vs. TBWF
62
23
38
c
SHR
TBWF
K07513
K00477
K07753
K13354
K08764
K17879
K04565
K13336
K13239
K11147
K00273
K04564
K11992
K11187
K13337
K00624
K03781
K13299
K12261
K00031
K11517
K05677
K13237
K00011
K00079
K01307
K01724
K00287
K15376
K20967
K03635
K00411
K00413
K00626
K02259
K03217
K03106
K00688
Peroxisome
Folate biosynthesis
Two-component system
Quorum sensing
Biofilm formation
Z Score
2
1
0
−1
−2
